# Conformational dynamics of RNA G_4_C_2_ and G_2_C_4_ repeat expansions causing ALS/FTD using NMR and molecular dynamics studies

**DOI:** 10.1093/nar/gkad403

**Published:** 2023-05-22

**Authors:** Amirhossein Taghavi, Jared T Baisden, Jessica L Childs-Disney, Ilyas Yildirim, Matthew D Disney

**Affiliations:** Department of Chemistry, Scripps Research and The Herbert Wertheim UF-Scripps Institute for Biomedical Research & Innovation, 130 Scripps Way, 3A1 Jupiter, FL 33458, USA; Department of Chemistry, Scripps Research and The Herbert Wertheim UF-Scripps Institute for Biomedical Research & Innovation, 130 Scripps Way, 3A1 Jupiter, FL 33458, USA; Department of Chemistry, Scripps Research and The Herbert Wertheim UF-Scripps Institute for Biomedical Research & Innovation, 130 Scripps Way, 3A1 Jupiter, FL 33458, USA; Department of Chemistry and Biochemistry, Florida Atlantic University, 5353 Parkside Drive, Jupiter, FL 33458, USA; Department of Chemistry, Scripps Research and The Herbert Wertheim UF-Scripps Institute for Biomedical Research & Innovation, 130 Scripps Way, 3A1 Jupiter, FL 33458, USA

## Abstract

G_4_C_2_ and G_2_C_4_ repeat expansions in chromosome 9 open reading frame 72 (*C9orf72*) are the most common cause of genetically defined amyotrophic lateral sclerosis (ALS) and frontotemporal dementia (FTD), or c9ALS/FTD. The gene is bidirectionally transcribed, producing G_4_C_2_ repeats [r(G_4_C_2_)^exp^] and G_2_C_4_ repeats [r(G_2_C_4_)^exp^]. The c9ALS/FTD repeat expansions are highly structured, and structural studies showed that r(G_4_C_2_)^exp^ predominantly folds into a hairpin with a periodic array of 1 × 1 G/G internal loops and a G-quadruplex. A small molecule probe revealed that r(G_4_C_2_)^exp^ also adopts a hairpin structure with 2 × 2 GG/GG internal loops. We studied the conformational dynamics adopted by 2 × 2 GG/GG loops using temperature replica exchange molecular dynamics (T-REMD) and further characterized the structure and underlying dynamics using traditional 2D NMR techniques. These studies showed that the loop's closing base pairs influence both structure and dynamics, particularly the configuration adopted around the glycosidic bond. Interestingly, r(G_2_C_4_) repeats, which fold into an array of 2 × 2 CC/CC internal loops, are not as dynamic. Collectively, these studies emphasize the unique sensitivity of r(G_4_C_2_)^exp^ to small changes in stacking interactions, which is not observed in r(G_2_C_4_)^exp^, providing important considerations for further principles in structure-based drug design.

## Graphical Abstract

**Graphical Abstract:**
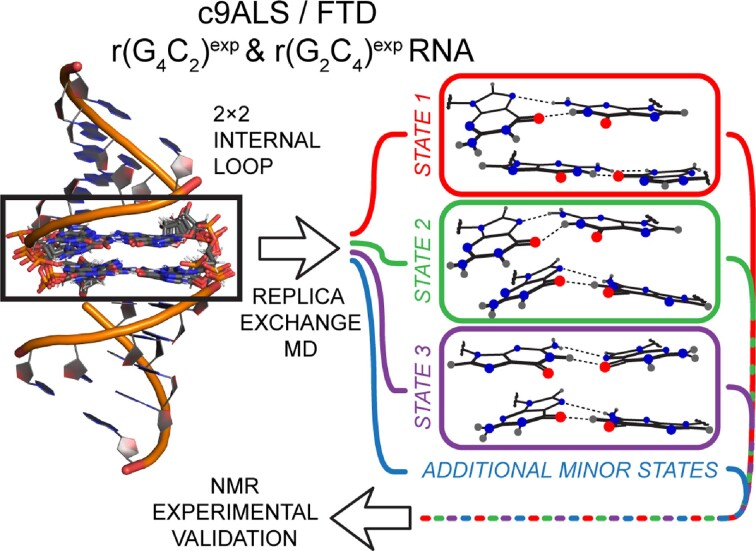

## INTRODUCTION

The inherently complicated conformational landscape of RNA includes a myriad of structural motifs such as pseudoknots, triple helices, multibranch loops, internal loops, bulges, and hairpins (1,2). A mounting body of evidence suggests that conformational transformations in RNA structures are often required for normal cellular function (3–5). Dynamic RNAs, however, pose a unique challenge to current experimental techniques used in structural biology in characterization of atomistic details. The conformational landscape of RNA encodes unique structures, which give rise to a diverse RNA structural ensemble (6). In addition, interactions with macromolecules such as proteins and small molecules can perturb the native conformations and alter the overall structure (7). Indeed, conformational dynamics discovered within RNA hairpin structures have recently been shown to play an important role in downstream biology with low-population states affecting biological outcomes (3,8,9). These ensembles offer both intriguing and unique targets for structure-based drug design (10). Collectively, these observations challenge the assumption that the global minimum conformation of the apo state is the primary target and suggest that conformations of local minima hold the key to improve selectivity while targeting RNA with small molecules (11,12).

The prerequisite of this structure-based lead-compound design (SBLD) approach is the understanding of the various conformations adopted by an RNA target. Interestingly, many disease-causing RNA molecules, especially those triggering neuromuscular diseases, include repetitive sequences of nucleotides that have been shown to adopt hairpin structures (13). These repeat expansions include trinucleotide repeats, which cause myotonic dystrophy (14) (DM), Huntington disease (HD) (15), fragile X syndrome (FXS) (16), and others (17). For these diseases, RNA internal loop motifs contained within the folded state has been shown to yield RNA gain-of-function. For example, expansion of CAG repeats found in Huntingtin gene translates into toxic polyglutamine (polyQ) proteins and sequesters proteins such as MBNL1 that cause HD. These internal loops, which vary in composition and size, sequester RNA-binding proteins involved in post-transcriptional gene regulation in foci either in nucleus or cytoplasm (18,19). Targeting these loops with small molecules can rescue various disease-associated phenotypes. Structural and modeling studies suggest that small molecules, which selectively target the loop regions in repeat expansions, can exploit these binding pockets (20–22). RNA internal loops have varying levels of conformational flexibility depending on the nucleotide composition and the neighboring base pairs. Residues in RNA internal loops can stack or unstack (stack ↔ unstack) from helical axis or rotate around the glycosidic bond (*anti* ↔ *syn*) that can stabilize the duplexes (23). Among all nucleotides, guanosine has the highest tendency to adopt the *syn* conformation (24). As a result, understanding the different conformations adopted by unique RNA loop motifs can help structure-based drug design.

**Figure 1.**
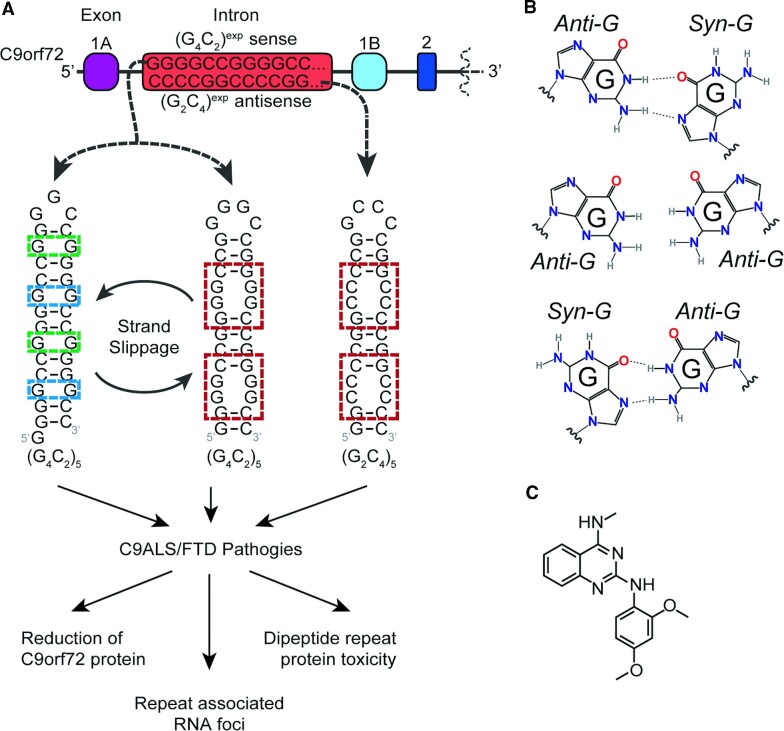
r(G_4_C_2_)^exp^ and r(G_2_C_4_)^exp^ as studied by NMR spectroscopy and MD simulations. (**A**) Secondary structures of the sense, r(G_4_C_2_)^exp^, and antisense, r(G_2_C_4_)^exp^, transcribed from C9orf72 can adopt different hairpin conformations. Note that 1 × 1 G/G ↔ 2 × 2 GG/GG transformations in r(G_4_C_2_)^exp^ results due to strand slippage. (**B**) Conformational states of 1 × 1 G/G internal loops observed in the literature. (**C**) Structure of small molecule CB253 (29), which stabilizes the 2 × 2 GG/GG internal loops in r(G_4_C_2_)^exp^.

The hexanucleotide repeat, r(G_4_C_2_)^exp^, harbored in intron 1 of chromosome 9 open reading frame 72 (C9orf72) mRNA, causes genetically defined amyotrophic lateral sclerosis (ALS) and frontotemporal dementia (FTD) (c9ALS/FTD) (25,26). C9orf72 mRNA is bidirectionally transcribed producing repeat expansions in both the sense, r(G_4_C_2_)^exp^, and antisense, r(G_2_C_4_)^exp^, strands that contribute to disease (Figure 1A). This r(G_4_C_2_)^exp^ adopts multiple structures, including G-quadruplexes (27) and hairpin structures (13), the latter of which contains an array of 1 × 1 G/G internal loop conformations (Figure 1B). It has been shown that 1 × 1 G/G internal loops are very dynamic and can interconvert between *syn*–*anti* and *anti*–*syn* (*syn*–*anti* ↔ *anti*–*syn*) (28).

Recently, we showed that a small molecule, CB253 (Figure 1C), targeting r(G_4_C_2_) hexanucleotide repeat expansions associated with ALS/FTD can cause strand-slippage to transform the global RNA structure to an alternative form, which has 2 × 2 GG/GG internal loops (29). This internal loop is both transient and highly dynamic, displaying rapid 1 × 1 G/G ↔ 2 × 2 GG/GG transformations that prevents its characterization by traditional biophysical techniques. ^19^F NMR studies utilizing ^19^F-modified cytidine residues in closing base pairs indicate that 1 × 1 G/G and 2 × 2 GG/GG internal loop motifs comprised ∼70% and ∼30% of the folding ensemble, respectively, in the absence of CB253 (Figure 1C) (29). Complementing these experiments with MD simulations using an RNA duplex model system showed the transition of two 2 × 2 GG/GG loops to three 1 × 1 G/G loops capturing the slippage phenomena. Although the outcomes of both experimental and computational study were in agreement, it should be noted that direct comparison of the percentage of each population from the two types of studies is not possible due to the differences of RNA systems used; experiments were completed on a single-stranded r(G_4_C_2_)_8_, which forms a hairpin structure, while predictions utilized the RNA duplex r(GGGGCCGGGGCC)_2_. *In vitro* experimental studies also showed that 2 × 2 GG/GG conformation was preferred by r(G_4_C_2_)^exp^ in the presence of CB253 (29). This observation provides a new direction in targeting r(G_4_C_2_)^exp^ with small molecules that can inhibit the pathobiological effects driven by this expanded repeat. Nevertheless, little is known about the properties of 2 × 2 GG/GG internal loops.

Compared to 2 × 2 GG/GG internal loops, 2 × 2 CC/CC internal loops, which can be observed in antisense r(G_2_C_4_)^exp^ strand, are not very dynamic. A recent structural study displayed that tandem C/C mismatches prefer all-*anti* orientations (30), which is in line with previous observations that pyrimidines are mostly observed in *anti* states (31). Nevertheless, circular dichroism (CD) and differential scanning calorimetry (DSC) studies on r(CCCCGG)_n_, where n was varied from 2 to 10, suggest also the existence of alternative structures (30), an indication that 2 × 2 CC/CC loops are not as rigid as suggested by X-ray studies. Because antisense RNA foci have been also observed in abundance in neurons of the frontal cortex (32), they can be exploited by small molecules targeting the 2 × 2 CC/CC loop motifs.

In this contribution, we expanded our previous studies and determined the structural ensemble adopted by 2 × 2 GG/GG and 2 × 2 CC/CC internal loops. Recent advances in NMR spectroscopy have provided the opportunity to validate the existence of transiently formed low populated conformations, adding to the repository of structural candidates that can be targeted by small molecules. Despite these advances, it is almost impossible to capture all the possible conformations via current experimental approaches. Therefore, we combined molecular dynamics (MD) calculations with NMR experiments, including the use of site-specific modified RNA residues, to determine the conformational states of 2 × 2 GG/GG and 2 × 2 CC/CC. Combined MD and NMR results display that 2 × 2 GG/GG internal loops in GGGC/GGGC observed in r(G_4_C_2_)^exp^ prefer *syn-anti*/*anti-syn* and *anti-syn*/*anti-syn* conformational states. Furthermore, while the C/C mismatches prefer single hydrogen bond states, we observed that the neighboring base pairs in 2 × 2 CC/CC internal loops affect the loop dynamics. For example, it was seen that CCCG/CCCG displays a single conformational state while two states were dominating GCCC/GCCC. Altogether, this study provides unique structural data on low populated conformations observed in folded states of r(G_4_C_2_)^exp^ and r(G_2_C_4_)^exp^, which can be utilized in structure-based drug design to specifically and effectively target 2 × 2 GG/GG and 2 × 2 CC/CC internal loops detected in C9orf72 transcripts.

## MATERIALS AND METHODS

### System preparation and molecular dynamics (MD) simulations

Simulations were carried out with the AMBER 18 (33) simulation package using the PARM99 force field (34) with revised *χ* (35) and *α*/*γ* (36) torsional parameters. The NAB (37) module of AMBER 18 was utilized to build the initial structures of r(5′-UCUGGGGCCAGA-3′)_2_, r(5′-UCUGGCCCCAGA-3′)_2_ and r(5′-UCUCCCCGGAGA-3′)_2_ in A-form RNA orientations, where underlined regions display the 2 × 2 internal loops. Each system was first neutralized with Na^+^ ions (38), which then was solvated with TIP3P (39) water molecules in a truncated octahedral box with periodic boundary conditions extending to 10 Å using the LEAP module of AMBER 18. The structures were minimized with the sander module each in two steps. Positional restraints on RNA heavy atoms with restraint weights of 10 kcal mol^−1^ Å^−2^ were applied in the first step of minimization with 2500 steps of steepest-descent algorithm followed by 2500 steps of conjugate-gradient algorithm. Second round of minimization was performed without restraints with 2500 steps of steepest-descent algorithm followed by 2500 steps of conjugate-gradient algorithm (Table S1).

Minimization was followed by an equilibration protocol first in constant volume dynamics, where positional restraints were imposed on the RNA heavy atoms with restraint weights of 10 kcal mol^−1^ Å^−2^ while temperature was gradually increased from 0 K to 300 K within several nanoseconds using the Langevin thermostat (Table S1). A second round of equilibration was performed at constant pressure (40), where temperature and pressure coupling were set to 300 K and 1.0 ps^−1^, respectively, while constraints on the solute were gradually removed (Table S1). After minimization and equilibration, MD simulation with a 2 ps time step was performed on each system using NPT dynamics with isotropic positional scaling. The reference pressure was set to 1 atm with a pressure relaxation time of 2 ps. SHAKE (41) was turned on for constraining bonds involving hydrogen atoms. An atom-based long-range cutoff of 10.0 Å was used in the production runs. The reference temperature was set to 300 K. PME was used to handle the electrostatics (42) and the Langevin thermostat (43) was applied with a coupling constant *γ* = 1.0 ps^−1^. Simulations were performed using the pmemd.cuda implementation of AMBER18.

### Temperature replica-exchange molecular dynamics (T-REMD) simulations

A simulated annealing protocol (44,45) was utilized with NMR restraints imposed on the RNA in 5′-UCUGGGGCCAGA-3′ to create the loop residues in all-*syn* orientations while keeping the global RNA structure in A-form orientation. The generalized Born implicit solvent model (46) with a 0.3 M NaCl concentration was used in simulated annealing. The system was first heated from 0 to 400 K for 5 ps and then cooled to 100 K. Force constants of 50 kcal mol^−1^ Å^−2^ and 50 kcal mol^−1^ rad^−2^ were used for distance and dihedral angle restraints, respectively to create a single structure at the end with loop residues in *syn*-*syn*/*syn*-*syn* configuration. This final structure, which displayed 2 × 2 GG/GG loop residues all in *syn*, was used as initial structure in T-REMD simulations. The initial structure was solvated and then equilibrated as described in the previous section.

For T-REMD simulations, for which restraints were not employed, 40 replicas spanning the temperature 277–374.5 K were used. A preliminary assessment with an exchange attempt at 10 ps with this temperature ladder resulted in exchange probability of 20–30%. Langevin thermostat with a collision frequency of 2 ps^−1^ was used to control the temperature in each replica. PME with 8.0 Å cutoff along with periodic boundary conditions and constant volume dynamics (NVT) was used in all T-REMD simulations (Table S1). Simulations were performed using pmemd.cuda.MPI of AMBER18. Each replica was run for 43 μs.

### Trajectory and cluster analyses

Trajectory analysis and sorting out the target temperatures were done with the CPPTRAJ module of AmberTools 18 (47). Analyses of the T-REMD were done at the 299.5 K replica, closest to the room temperature. Cluster analysis was completed using the average-linkage hierarchical agglomerative method (48). Heavy atoms of 2 × 2 GG/GG loop residues were used in the cluster analyses with a root-mean-square deviation (RMSD) of 1.2 Å. We also used an in-house script, as described previously, which incorporates symmetry in the cluster analyses. This second clustering method, which used an RMSD value of 1 Å, was used to cluster similar snapshots from the T-REMD trajectory (Script S1). All the predicted clusters are based on this second method. While analyzing the T-REMD trajectory performed on 2 × 2 GG/GG, only the clusters observed over 1% are further analyzed. To test if the calculated percentages are converged in the T-REMD trajectory, an in-house code (49) (Script S2) was utilized. The first 10 μs was discarded. Percentages for each cluster along the MD trajectory covering 500 ns were calculated at every time step.

### Kullback-leibler divergence and cluster percentage fluctuation analysis

CPPTRAJ was used for principal component (PC) and Kullback–Leiber (KL) divergence analyses. As explained in reference (50), time dependent KL divergence is calculated as:


$$\begin{eqnarray*}KLD\,\left( t \right)\, = \sum\limits_i {P\left( {t,i} \right)} \,\ln \left( {\frac{{P\left( {t,i} \right)}}{{Q\left( {t,i} \right)}}} \right)\end{eqnarray*}$$

where $P( {t,i} )$ and $Q( {t,i} )$ represent the probability distributions of data sets $P$ and $Q$ and $i$ is the histogram bin index which includes all the data from time 0 to $t$. The last 10 μs of the T-REMD simulation was divided into two 5 μs long trajectories (30–35 and 35–40 μs) to assess the convergence of the simulations. In this approach, we assumed these two 5 μs long trajectories as two independent simulations. In the process, the covariance matrix was calculated for the 10 μs long trajectory so that each frame was first RMS-fit to an average structure to remove the rotational and translational motions. The eigenvalues and eigenvectors were then obtained for each 5 μs trajectory through diagonalization of the covariance matrix. Consequently, coordinates from each 5 μs trajectory were projected along eigenvectors of choice to obtain the projected values for each given mod. A Gaussian kernel density estimator with 300 bins was utilized to create a histogram for each data set, where Kullback-Leibler divergence at each frame was calculated. The sum over each histogram was then normalized to 1.0 (50). The script used for this analysis is provided in SI (Script S3).

### Preparation of the NMR samples

RNA oligonucleotides of r(CCAGGGCAAG**GAAA**CUUGGGCUGG) (where loop nucleotides are underlined and the GAAA tetraloop is bold), including the m^1^G modified ones, were purchased from Dharmacon (GE Healthcare) as 2′ACE-protected oligonucleotides. Samples were deprotected according to the manufacturer's protocol followed by desalting with a PD-10 Sephadex column (GE Healthcare). RNA concentration was quantified by its absorbance measured at 260 nm and 90°C using a Beckman Coulter DU-800 UV/vis spectrophotometer and the corresponding extinction coefficient provided by the vendor. An aliquot of 10 × NMR Buffer in H_2_O (100 mM Na_2_HPO_4_, pH 6.0, and 1 mM EDTA) was added to each sample to a final concertation of 1 × NMR Buffer and 600 μM RNA.

To study exchangeable protons, D_2_O was added to achieve a ratio of 5% D_2_O, 95% H_2_O. For non-exchangeable protons, samples were prepared, evaporated to dryness and subsequently redissolved in the same volume of 99.996% D_2_O (Sigma). After preparation, samples were heated to 90°C for 3 min and cooled on ice before adding to NMR tubes. Dilution titrations were performed by removing aliquots from 2D samples, adding appropriate amounts of NMR buffer, and refolding again before completing additional 1D NMR experiments.

### 1H NMR spectroscopy

All NMR experiments were performed on Bruker Avance III 700 MHz and 600 MHz spectrometers equipped with 5-mm triples resonance (TCI) cryogenic probes. 1D and 2D imino (exchangeable) protons were recorded in 5% D_2_O 95% H_2_O at 278°K (5°C) for unmodified 2 × 2 GG/GG and 2 × 2 CC/CC NMR constructs using the 600 MHz spectrometer. All other experiments were performed on the 700 MHz spectrometer. The m^1^G-labeled samples in H_2_O were recorded at 283 K (10°C). 2D NOESYs for non-exchangeable protons were recorded at 298°K (25°C) using a mixing time of 300 ms. Assignments were obtained using standard 2D NOESY, DFQ-COSY and ^1^H–^13^C HSQC Bruker experiments with excitation sculpted water suppression. All spectra were processed and analyzed using TOPSPIN 4.0.6 (Bruker) and visualized using Sparky 3.115 (University of California, San Francisco) (51).

## RESULTS

### Validation of the RNA force field - slippage phenomena in r(G_4_C_2_)^exp^

Our previous studies as well as those completed by others report that depending on the salt properties r(G_4_C_2_)^exp^ prefers either a hairpin structure with repeating 1 × 1 G/G internal loops in *syn*-*anti* orientations (Figure 1A, B) or a G-quadruplex structure (52). Moreover, a small molecule, CB253, (Figure 1C) revealed formation of a minor and highly dynamic RNA structure, where the repeating 1 × 1 G/G loops transformed to 2 × 2 GG/GG due to strand slippage, which was detected by NMR spectroscopy (29). Before investigating the structural properties of 2 × 2 GG/GG internal loops using T-REMD, we validated the RNA force field.

The accuracy of computational predictions depends on the quality of the force field. Compared to protein force fields, development and fine tuning of RNA force fields is lagging (53–56). We, therefore, first benchmarked commonly used RNA force fields to assess their accuracies. There have been various attempts to improve the accuracy of RNA force fields mainly through the refinement of torsional parameters (35,53,57–59). Currently four RNA force fields are available within the AMBER suite, including: (i) OL3 (59,60), (ii) Rochester (58), (iii) DE.Shaw (57) and (iv) RNA.YIL (36). Previously, we showed that a slippage phenomenon occurs in the context of r(G_4_C_2_)_2_ through 70 μs long MD simulations using the RNA.YIL force field with revised *α*/*γ* torsional parameters (dubbed RNA.YIL.AG)(37). This force field was able to recapitulate experimental observations where 2 × 2 GG/GG loops are short lived and convert to 1 × 1 G/G loops, as observed in apo form of r(G_4_C_2_)_4_(29).

**Figure 2.**
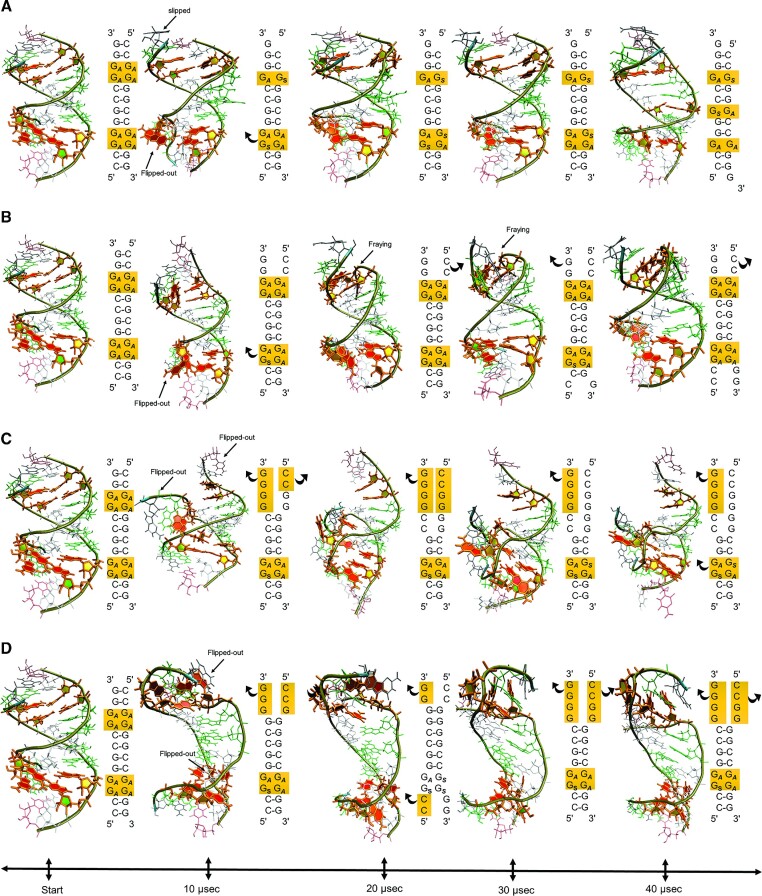
Evaluation of four different RNA force fields in recapitulating strand slippage in model r(G_4_C_2_)_2_. (**A**) RNA.IL.AG is the only force field among the benchmarked force fields, which maintains the RNA helicity while transforming two 2 × 2 GG/GG to three 1 × 1 G/G loops via strand slippage. (**B**) OL3 force field could maintain the helicity of the RNA along the MD simulation but couldn’t reproduce the slippage. Simulations were stopped after nearly 42 μs. (**C**) DE.Shaw force field is unable to maintain the helicity of the RNA after 5 μs, and the distorted construct remained unchanged till the end of MD simulation (45 μs). (**D**) Rochester force field performs better than DE.Shaw but the misfolded structure was maintained along the simulation and no *anti* → *syn* transformation was observed (see Movie S1). ***A*** and ***S***indicate *anti* and *syn* configurations, respectively.

We applied all four force fields to assess whether each could reproduce strand slippage in a model duplex, r(G_4_C_2_)_2_. The secondary structure of the model RNA at the beginning of the simulations was 5′-GGGGCCGGGGCC-3′, where the underlined regions represent the 2 × 2 GG/GG loops. A 40 μs long MD simulation for each force field showed that, with the exception of RNA.IL.AG, they were unable to reproduce the slippage phenomenon. As previously observed, the RNA.IL.AG force field showed the conversion of two 2 × 2 GG/GG loops into three 1 × 1 G/G loops (29). During this process, fraying of terminal base pairs as well as guanosine residues flipping out from the helical axis were observed (Figure 2A, Movie S1). The flipping-out of the guanosine residues, however, did not perturb the overall helical structure of the RNA, as they stacked back inside the helical axis. After the completion of the slippage event, the RNA construct formed three 1 × 1 G/G loops in *anti-syn*, *syn-anti*, and *anti-syn* orientations respectively. To ensure the observed orientations for three 1 × 1 G/G loops are stable, this simulation was extended to cover over 70 μs for the RNA.IL.AG. The MD simulations of the other three force fields were stopped after 40 μs as none of them were able to reproduce the slippage phenomenon and thus the 2 × 2 GG/GG → 1 × 1 G/G transformations (Movie S1).

In the first 10 μs of the MD simulation completed by the OL3 force field, no major structural distortions were observed, and the helicity of the RNA construct was conserved (Figure 2B). Between the 10–15 μs interval, fraying of terminal bases were observed including loss of hydrogen bonds in terminal GC base pairs. Between 15 and 20 μs, slippage at one terminal occurred, creating a 1 × 1 G/G loop in the *anti*-*syn* orientation while the distortion at the other terminal remained intact, where this conformational state was maintained until 30 μs. After this point on fraying of terminal base pairs was observed. Even though the OL3 force field did not display a transformation from 2 × 2 GG/GG to 1 × 1 G/G loops, it is possible that the 2 × 2 GG/GG state is too stable in OL3 and as a result would require more time to observe the desired conformational change. As extensive MD simulation time would be required to exit this local minimum entrapment and reach the global minimum, we stopped the simulations after this point. In the simulation by the DE.Shaw force field, the helicity of the RNA duplex was mostly deteriorated within the first 5 μs, with several terminal base pairs fraying (Figure 2C). Continuation of the simulation generated a highly distorted structure. This misfolded conformation remained unchanged until the end of the simulation with a total MD time of 40 μs. Within 2 μs of simulations using the Rochester force field, base pairing in terminal base pairs was lost (Figure 2D). RNA structure was distorted from this point on until the end of the MD simulation. After benchmarking four RNA force fields and showing that only RNA.IL.AG reproduced the slippage phenomena, we utilized this RNA force field in the T-REMD calculations.

### Extensive sampling done by T-REMD displays unique conformations adopted by 2 × 2 GG/GG

Conformations of RNA 1 × 1 G/G loop motifs have been extensively studied as compared to 2 × 2 GG/GG loops. Using the RNA CoSSMos database (61), we initially extracted all the structures of 1 × 1 G/G internal loops observed in the literature. Analyses showed that 1 × 1 G/G loop structures adopt *anti*-*syn*, *syn*-*anti*, and *anti*-*anti* orientations, comprising 55%, 16%, and 26% of structures, respectively (Figure 1B and Table S2). In contrast, the configurational preferences of 2 × 2 GG/GG internal loops have not been studied greatly as evidenced by the lack of structures available in the literature. The highly dynamic nature of 2 × 2 GG/GG internal loops is likely one of the reasons why structural studies have not been reported. Inspired by the discovery of a small molecule, CB253, (Figure 1C) which reorganizes the conformation of r(G_4_C_2_)^exp^ to adopt 2 × 2 GG/GG internal loops rather than 1 × 1 G/G, we investigated the conformational landscape adopted by 2 × 2 GG/GG internal loops using both NMR methods and MD calculations.

**Figure 3.**
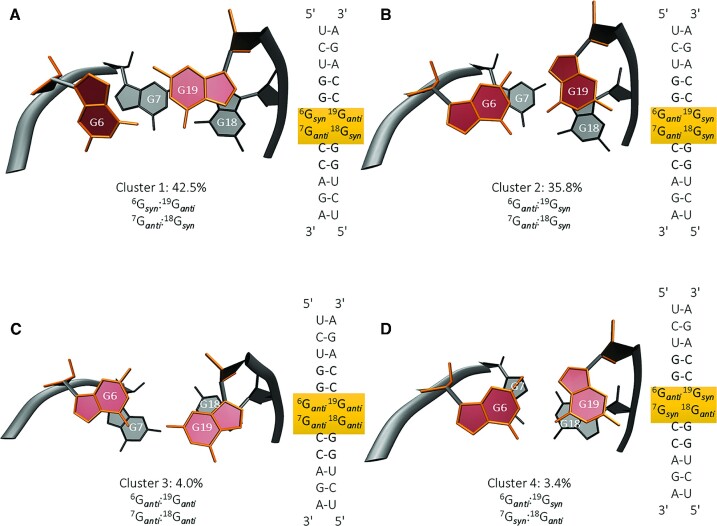
Four of the most observed clusters after analyzing the T-REMD ensemble (at 299.5 K) of the model 2 × 2 GG/GG loop system. (**A**) *syn*–*anti*/*anti*–*syn*, (**B**) *anti*–*syn*/*anti–syn*, (**C**) *anti–anti*/*anti–anti* and (**D**) *anti–syn*/*syn–anti*. An in-house script (Script S1) incorporating symmetry of the studied system with an RMSD of 1.0 Å was utilized to cluster the snapshots observed in T-REMD trajectory. For better recognition of different orientations adopted by the 2 × 2 GG/GG loop figures are shown in top view.

To scan the conformational landscape of 2 × 2 GG/GG internal loops, we employed an advanced computational sampling method, temperature replica exchange MD (T-REMD). In T-REMD, a series of replicas of the same system are created, each running at a different temperature with exchange attempts periodically done between replicas. As implemented in AMBER 18, the acceptance or rejection of exchange attempts is performed using the Metropolis Monte Carlo criterion such that the frequencies of the observed conformations approach to the equilibrium conditions, within the limits of substantial number of simulation steps (62,63). A model RNA, r(UCUGGGGCCAGA)_2_, was utilized for this purpose, where internal loop residues are underlined to highlight the 2 × 2 GG/GG motif. At the start of the simulations, all the guanosine loop residues were modeled to be in *syn* orientations, r(UCUGGG_*syn*_G_*syn*_CCAGA)_2_, which displays the least stable conformation adopted by 2 × 2 GG/GG residues, thereby facilitating conformational transitions and avoiding the system to be trapped in local minima. No restraints were used for these simulations. As noted above, CoSSMos database did not display any *syn*-*syn* orientations for 1 × 1 G/G mismatches (Table S2), which rationalizes our choice of the initial structure, which has all loop residues in *syn*. We used 40 replicas each running for 43 μs with a cumulative time of ∼1.7 ms. We then performed cluster analyses to assess the different configurations adopted by the 2 × 2 GG/GG loop and calculated the observed percentages for each cluster. Over a dozen of clusters with different orientations were identified with two major configurations comprising more than 78% of the total population (Figure 3). The major configuration adopted by the 2 × 2 GG/GG loop was G_*syn*_G_*anti*_/G_*anti*_G_*syn*_ observed 42.5% of the population (Figure 3A) followed by G_*anti*_G_*syn*_/G_*anti*_G_*syn*_ observed 35.8% (Figure 3B). Two minor clusters were also observed adopting G_*anti*_G_*anti*_/G_*anti*_G_*anti*_ and G_*anti*_G_*syn*_/G_*syn*_G_*anti*_ at 4% and 3.4%, respectively (Figure 3C, D).

### Assessing the convergence of the T-REMD results

**Figure 4.**
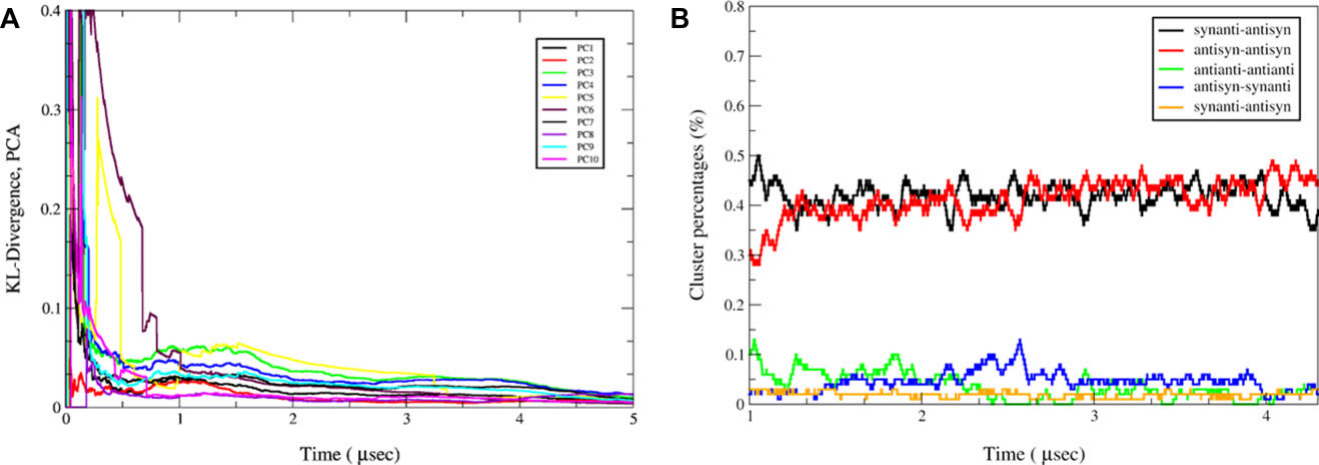
Convergence analyses performed on T-REMD ensemble at 299.5 K. (**A**) Kullback−Leibler divergence (KLD) of principal component projection for the first 10 principal components between 30–35 μs and 35–40 μs of the T-REMD trajectory. (**B**) Monitoring the percentage changes of the highest observed five clusters extracted from 43 μs long T-REMD simulations. The first 10 μs of the simulations have been discarded in (B). Note that calculated percentages at each time step analyzes 500 ns MD trajectory. Note also that the percentages displayed in black, red, green, and blue are calculated with respect to the predicted clusters of Figure 3A, B, C and D, respectively.

To assess the rate of convergence, Kullback−Leibler divergence (KLD) analysis was performed (50). KLD is a statistical analysis approach, which measures the difference of one probability distribution with another probability distribution serving as the reference. In this approach the overlap of the principal component (PC) projection histograms is used as a measure of convergence between two independent runs. Therefore, if these two runs experience the same motions and sample the same conformational space, the overlap of their PCs increases. A well converged simulation is the one in which PC projection histograms have a good overlap. Figure 4A shows the KLD of the first ten principal components for the simulation intervals 30–35 and 35–40 μs. It was observed that KLD values drop to ∼0.03 after 3 μs and remain unchanged implying a well converged simulation. We applied the same convergence analysis to the intervals 20–25 and 25–30 μs (Figure S1). Although the changes of KLD values are not as low as observed for the intervals displayed in Figure 4A, both analyses still show very good convergence, especially with respect to the first three PCs.

In order to verify the results of KLD analyses, we conducted another type of convergence analysis, where the populations of each cluster observed along the trajectory was calculated (Figure 4B). After performing cluster analyses on the T-REMD ensemble at 299.5 K, clusters observed over 1% were first determined. Observed percentages of these clusters along the T-REMD trajectory were then calculated at every time step covering 500 ns snapshots (Figure 4B). It was observed that the two major clusters, G_*syn*_G_*anti*_/G_*anti*_G_*syn*_ and G_*anti*_G_*syn*_/G_*anti*_G_*syn*_, were the dominant ones fluctuating around 40%, which were reasonably well with the predictions displayed in Figure 3.

### Studying the structures of 2 × 2 GG/GG internal loops by NMR spectroscopy

**Figure 5.**
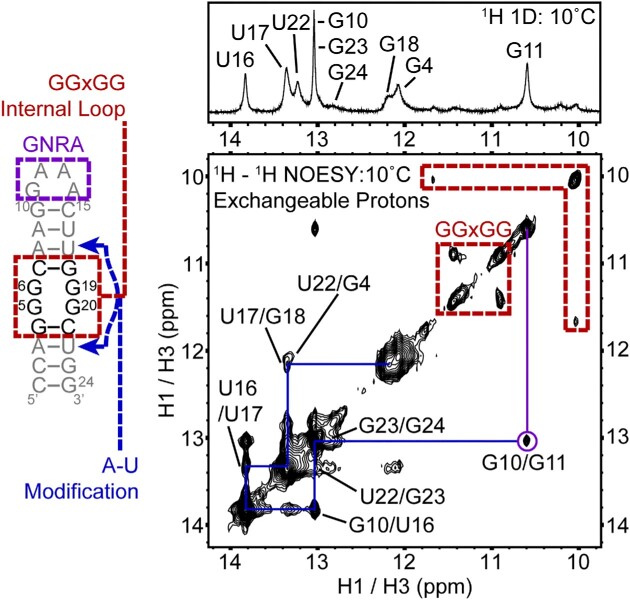
Model RNA utilized to investigate structural properties of 2 × 2 GG/GG internal loops using NMR spectroscopy. Secondary structure of the model RNA containing the 5′-GGGC-3′/3′-CGGG-5′ internal loop found in r(G_4_C_2_)^exp^ (left) and its ^1^H–^1^H imino NOESY spectrum (right). Both the RNA and spectrum are color coded as follows: the GNRA tetraloop in purple; upper and lower stems of the hairpin in blue, loop nucleotides in red, and assignments in gray.

To verify the computational predictions, the RNA construct, r(CCAGGGCAAGGAAACUUGGGCUGG), was designed to eliminate potential strand slippage as observed previously (29). When folded, this construct includes a 2 × 2 GG/GG internal loop as well as a stable GNRA tetraloop of GAAA, a site-specific G/C to A/U modification adjacent to the closing pairs of the internal loop, and two capping GC base pairs (Figure 5). Preliminary ^1^H 2D NMR characterization of exchangeable protons revealed a well-formed GNRA tetraloop, with an off-diagonal peak at ∼10.5 ppm corresponding to stacking between the GNRA and upper helix (64) (Figure 5). The canonical base pairs of the upper and lower helix are well formed, with sharp peaks in the 12.5–14.0 ppm range. A 2D nuclear overhauser effect spectroscopy (NOESY) spectrum highlights neighboring base pairs observed by NMR, with blue lines referencing off-diagonal peaks, along with corresponding assignments (Figure 5). Interestingly, off-diagonal peaks for nucleotides G4 and G18 are broadened due to exchange processes of the neighboring internal 2 × 2 GG/GG internal loop nor do they have NOEs to these internal loop resonances (Figure 5). In addition, there are two sets of off-diagonal peaks in the non-canonical region that do not have NOEs to either the upper or lower helices, which are highlighted with red boxes and appear between 10 and 12 ppm (Figure 5). We suspected that these peaks corresponded to specific structure(s) in the 2 × 2 GG/GG internal loop but could not make definitive assignments due to exchange broadening of these dynamic nucleotides observed in the NMR spectra (Figure 5).

To confirm that the non-canonical peaks in these 2D experiments were the result of the 2 × 2 GG/GG internal loop and not a product of stem-stem interactions, we also performed a dilution experiment, where the RNA sample was diluted in buffer and refolded followed by acquiring the 1D spectrum (Figure S2A). In these experiments, there were no detectable changes upon dilution from 600 μM down to 50 μM, suggesting that these peaks arose from nucleotides in the 2 × 2 GG/GG internal loop and not from RNA-RNA interactions. In addition, the aromatic and ribose regions of the 1D spectrum showed clear, sharp peaks, confirming no aggregation or G-quadruplex formation at the highest concentration studied (Figure S2B).

To further our investigation of this model NMR construct, we completed non-exchangeable NMR experiments, including ^13^C-^1^H heteronuclear single quantum coherence spectroscopy (HSQC), ^1^H–^1^H 2D NOESY, and double quantum filtered correlation spectroscopy (DFQ COSY) experiments (Figure S3), which are not limited by solvent exchange associated with imino proton experiments (65). These experiments also revealed extensive broadening due to exchange with nucleotides within and neighboring of the 2 × 2 internal loop, broadening beyond detection. Nevertheless, the upper and lower stems neighboring the internal loop were helical, as shown by a complete NOESY-walk (Figure S3D and Table S3), interrupted only by the 2 × 2 GG/GG internal loop. As expected, initial results display that multiple conformational states are preferred by the 2 × 2 GG/GG internal loop in this NMR construct, which were observed at two temperatures (5°C and 10°C) and two NOESY mixing times (75 ms and 300 ms) (Figure S4A-C, S5A). In order to discover the specifics of the preferred states of 2 × 2 GG/GG internal loop in this construct, we performed site-specific m^1^G mutation studies (*infra vide*).

### Methyl-1 guanosine (m^1^G) mutations reveal two states dominate the conformational ensemble

**Figure 6.**
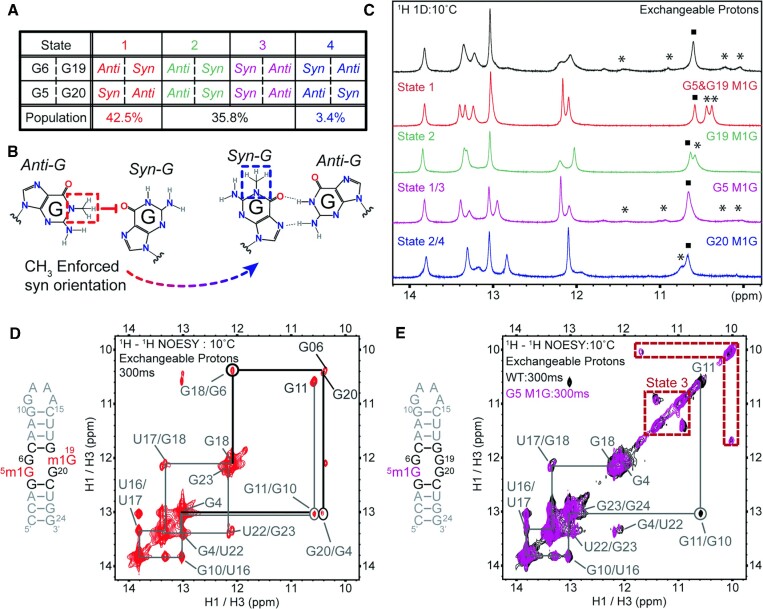
Deconvolution of the conformations adopted by 2 × 2 GG/GG internal loops using m^1^G modified constructs and NMR spectroscopy. (**A**) Structures extracted from in T-REMD simulations, including orientations and predicted populations for each state. (**B**) Structure of the 1-methylguanosine (m^1^G) modification, which prevents the G from adopting an *anti* conformation and enforces the *syn* conformation. (**C**) ^1^H 1D imino NMR spectrum of m^1^G mutants and their corresponding stabilized states, GNRA imino resonance denoted with ‘$\blacksquare$’ and non-canonical peaks denoted with ‘*’. (**D**) Secondary structure of the m^1^G5/m^1^G19 modified RNA, which adopts State 1 described by T-REMD in (A), and the corresponding ^1^H–^1^H imino NOESY spectrum. Signals from the GNRA tetraloop are circled; additional off-diagonal peaks for the lower and upper stem are shown as grey lines; non-canonical peaks associated with the 2 × 2 GG/GG are shown with thick black lines and lettering; and assignments for all other resonances shown in grey. (**E**) Secondary structure of the m^1^G5 modified RNA, which adopts State 1 and State 3 described by T-REMD in A and the corresponding ^1^H–^1^H imino NOESY spectrum (purple) overlaid on the spectrum from the unmodified RNA (black). Signals from the GNRA tetraloop are circled, additional off-diagonal peaks for the lower and upper stem are shown as grey lines; non-canonical peaks associated with the 2 × 2 GG/GG are shown with a red dashed line; and assignments for all resonances are shown in grey.

To validate the orientations of the internal loop residues predicted by T-REMD calculations (Figure 6A), we performed NMR studies on several singly and doubly modified RNA samples. We selected m^1^G modifications, which inhibit hydrogen bond donation from modified nucleotides (Figure 6B). Using this modification in the context of the 2 × 2 GG/GG internal loop, we anticipated that m^1^G-modified nucleotides will adopt the *syn* conformation, where the Hoogsteen face of the modified guanosine base pairs with the Watson-Crick side of the unmodified guanosine located on the opposite strand (Figure 6B). Modifications of G5 and G19 folds the RNA construct into the predicted most stable state, G_*syn*_G_*anti*_/G_*anti*_G_*syn*_, as predicted by T-REMD simulations and as evidenced by the sharp line width in the imino region of the 1D NMR spectrum (Figure 6C). Furthermore, two peaks, which form due to non-canonical base pairs denoted with ‘*’, appeared around 10.2 ppm, presumably due to stabilization of the 2 $ \times$ 2 GG/GG internal loop (Figure 6C, D). Peaks in the region where canonical base pairs appear were also sharp between 12 and 14ppm, indicating that this RNA adopted a single folded conformation in solution (Figure 6C, D). This state was further characterized by 2D imino NMR experiments, affording a complete NOESY walk for resonances in the upper and lower helices as well as the 2 × 2 GG/GG internal loop (Figures 6D and S5B). Completed assignments are shown, which reveal the stability of State 1 in this sequence context, showing off-diagonal peaks between the 2 $ \times$ 2 GG/GG and the upper and lower stems (Figures 3A and 6A, D).

Incorporation of a single m^1^G at position 19 enforced this nucleotide to adopt *syn* conformation, consistent with states 1 and 2 predicted by T-REMD (Figures 6A,B), with two peaks around 10.6 ppm and denoted with ‘*’ shifted upfield in 1D imino spectrum (Figure 6C), consistent with the formation of the GNRA tetraloop, denoted as ‘$\blacksquare$’ and a single non-canonically paired G/G. 2D imino NMR experiments (Figure S5C) displayed off-diagonal peaks mapping to the upper stem through the top of the 2 $ \times$ 2 GG/GG, with breaks in the NOESY-walk at the G5/G20 resonances. In this modified sequence, significant overlap between the two imino resonances G6 and G5/G20, which are attributed to the 2 $ \times$ 2 GG/GG internal loop, prevented the observation of any off-diagonal peaks.

We then performed NMR studies on another single modified mutant, m^1^G on position 5 (Figures 6C, E and S5D). This modification afforded a very similar spectrum to the unmodified system, with a single non-canonical peak appearing around 10.6 ppm, consistent with the formation of the GNRA tetraloop (Figure 6C). In addition, lower intensity peaks denoted with ‘*’ appeared at ∼10.0, 10.3 and 11.0 ppm in the non-canonical region as G_*syn*_/G_*anti*_ can be seen both upfield and downfield of this GNRA peak, although these resonances are significantly broadened by NMR exchange (Figure 6C). Characterization of this construct by 2D NMR studies revealed two sets of non-canonical peaks, which overlay with the NMR results of the unmodified RNA (Figure 6E). The remarkable match in both peak intensity and location conclusively shows that this singly modified construct has a ‘near’ identical conformational ensemble to the unmodified construct. The presence of two sets of non-canonical peaks in the NOESY spectrum collected at a longer mixing time (300 ms) confirmed that both states 1 and 3 predicted by T-REMD are the most stable states in solution (Figure 6A, E). However, it is important to note that the 1D spectrum for the unmodified RNA differed slightly from the m^1^G position 5 mutant, presumably due to exchange processes related to the other two states that are possible in the unmodified RNA (Figure 6C).

For the sake of completeness, we also subjected another singly modified RNA construct to NMR studies, one that recapitulates both states 2 and 4. Modification of G20 resulted in the most exchanged broadened spectrum of all m^1^G constructs, with significant broadening occurring at ∼10.6 ppm (non-canonically paired nucleotides), 12 ppm, and 13.2 ppm (Figure 6C). However, it is important to note that the GNRA tetraloop remained well formed, with a sharp, albeit overlapping peak, appearing at 10.6 ppm. When subjected to 2D NMR studies (Figure S5E), no off-diagonal peaks were observed between the lower stem, G4:G19, and the bottom of the modified 2 $ \times$ 2 GG/GG in ternal loop (G5/m^1^G20). This spectrum differs from the other singly-modified constructs, where both m^1^G19 and m^1^G5 residues, within the internal loop, had off-diagonal peaks with the neighboring upper or lower stems, which is consistent with stable formation of base pairs when modified nucleotides were utilized (Figure S5C–E). These differences in stability reveal that attempts to enforce G5 to adopt the *anti*-conformation (Figure 6A, states 2 and 4) are disfavored. In this instance, these states are rapidly interconverting, causing exchange broadening in the NMR spectra that makes these states undetectable by traditional 1D and 2D imino NMR methods. This conclusion is consistent with the m^1^G5 modified RNA, which enforces G5 to *syn* conformation and very closely resembles the NMR spectrum of the unmodified RNA (Figures 6E and S5D).

Together, these NMR studies confirmed that the G5 *syn* conformation was the most stable state, in agreement with T-REMD simulations (Figure 6A, states 1 and 3). Concurrently, this data also revealed that the NMR signals for G6:G19 base pair was broadened due to rapid interconversion between *syn* and *anti* states in all the constructs, including those with the m^1^G modifications as evidenced by exchange broadened NMR spectra (Figures S5C–E). The observable differences in dynamics between the G/G base pair neighboring the lower stem (G5:G20) and the G/G base pair neighboring the upper stem (G6:G19) were not expected, as the internal loop and neighboring nucleotides are symmetric. However, there was potential for energetic differences in stacking between the lower and upper stems based off: (i) A/U flanking both the 5′ and 3′ the internal loop causing asymmetry between the strands 5′-AGGGCA-3′ versus 5′-UGGGCU-3′ and (ii) the contribution of a 3 base pair stem versus a 3 base pair stem and hairpin for the bottom and top, respectively (Figure 5). To interrogate the contribution of further neighboring nucleotides on the 2 × 2 GG/GG loop dynamics, we designed an additional construct by swapping the base pairing location of the A-U pair in the upper stem (A8/U17 to U8/A17, termed ‘AU-swap’). This construct improved symmetry, with 5′-AGGGCU-3′ for both strands. NMR studies confirmed that this construct was highly symmetric (Figure S6), with resonances for the upper and lower stem highly overlapped due to the similarity in their local chemical environment and resulting NMR chemical shifts. Non-canonical peaks, again appeared in the 10–11 ppm region, with two distinct sets of off-diagonal peaks in the NOESY highlighted with a red box that represent imino signals for the 2 × 2 GG/GG loop. Assignments and characterization were not possible due to such highly overlapped peaks and will remain the subject of future studies. The resulting changes in 2D NMR spectra after a small change in nucleotide location confirmed that the 2 × 2 GG/GG internal loop is sensitive to stacking interactions and suggests that small changes in stacking energies may propagate into differences in dynamics observed between the two non-canonical G/G pairs of the 2 × 2 GG/GG internal loop.

### Closing base pairs affect the stability of 2 × 2 CC/CC internal loops formed by r(G_2_C_4_)^exp^

**Figure 7.**
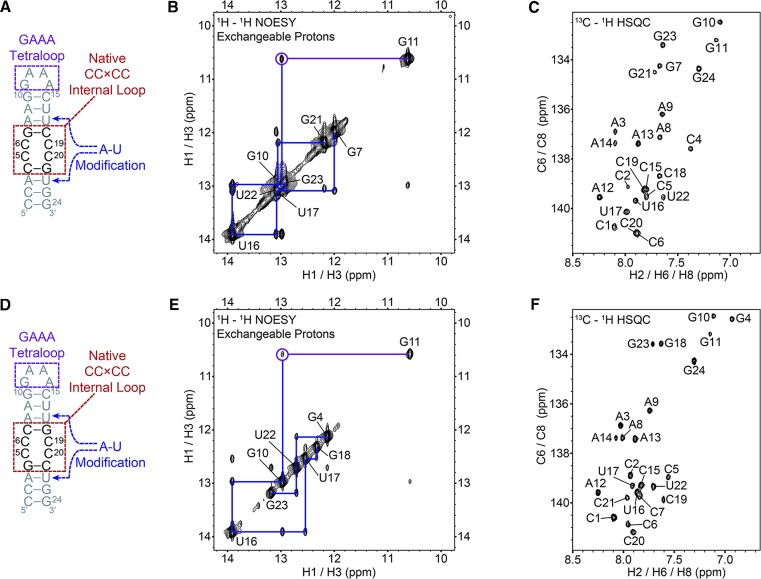
Model RNA constructs to investigate structural properties of 2 × 2 CC/CC internal loops formed by r(G_2_C_4_)^exp^ using NMR spectroscopy. Model RNA constructs to investigate C_4_G_2_ (CCCG) (**A**) and G_2_C_4_ (GCCC) (**D**). ^1^H–^1^H imino NOESY experiments of A (**B**) and D (**E**), with GNRA highlighted with a purple line and circle, additional off-diagonal peaks for the lower and upper stem shown as blue lines, and assignments for all resonances shown in gray. (C) ^13^C–^1^H HSQC experiments of A (**C**) and D (**F**) showing aromatic region with complete assignments.

In ALS/FTD, foci are composed of both sense r(G_4_C_2_)^exp^ and antisense r(G_2_C_4_)^exp^ RNAs (66), which can be targeted by small molecules for therapeutic uses. The antisense r(G_2_C_4_)^exp^ is a C-rich RNA sequence, which forms 2 × 2 CC/CC internal loops. At low pH, C-rich RNA sequences can form a four-stranded structure named RNA i-motif, which are not stable at physiological conditions (67). On the other hand, it is suggested that C-rich sequences would form excess non-Watson-Crick C/C base pairs (30). In an attempt to provide structural information, the antisense RNA structure of the *C9orf72* has been studied by X-ray crystallography, which displayed each C/C mismatch to form a single hydrogen bond in 2 × 2 CC/CC loops (30). This pattern of hydrogen bonding has been already observed in 1 × 1 C/C internal loops (68,69). The overall RNA structure was classified as A-form although multiple deviations from a typical A-form RNA was reported. Further studies on r(CCCCGG)_*n*_(CCCC) (2 ≤ *n* ≤ 10) using CD and DSC confirmed the existence of noncanonical base pairs possibly caused by strand slippage, which was also observed in X-ray constructs incorporating CCG repeats (68). Nevertheless, the fact that the crystal structure of a model r(CCCCGG)_3_(CCCC) was determined suggests that 2 × 2 CC/CC is less dynamic than 2 × 2 GG/GG (30). Having all these information available, we decided to study the 2 × 2 CC/CC loops using NMR spectroscopy and MD calculations. The antisense repeats, r(G_2_C_4_)^exp^, is predicted to form two types of 2 × 2 CC/CC internal loops: (i) 5′-CCCG-3′/5′-CCCG-3′ (CCCG) and(ii) 5′-GCCC-3′/ 5′-GCCC-3′ (GCCC). As a result, both of these systems were investigated to describe the structural details of 2 × 2 CC/CC internal loops formed by r(G_2_C_4_)^exp^. The same GNRA tetraloop described above, which was used to study 2 $ \times$ 2 GG/GG internal loops, was utilized to probe 2 × 2 CC/CC internal loops (Figure 7). The 5′-CCCG-3′/5′-CCCG-3′ sequence was flanked with neighboring AU base pairs to prevent strand slippage and eliminate spectral overlap to aid in NMR assignments (Figure 7A). In addition, a GNRA tetraloop cap was incorporated to enable distinct assignments for both the top and bottom helices (Figure 7A).

For the CCCG RNA, NMR studies revealed a well folded top and bottom helix, with a characteristic peak at ∼10.6 ppm consistent with the formation of the GNRA tetraloop (Figure 7B). Of note, cytosine residues lack imino protons and hence are not observable in 2D imino proton spectra. However, residues neighboring the 2 × 2 CC/CC internal loops, G7 and G21, had sharp line widths and were well resolved, indicating a stable internal loop within the neighboring helical context (Figure 7B). To study the dynamic nature of the loops, we completed both ^1^H-^13^C HSQC and ^1^H -^1^H D_2_O NOESY experiments (Figures 7C, S7, and Table S4). We also characterized the GCCC RNA by NMR (Figure 7D–F). In the 2D imino proton spectrum, the GCCC also appeared to fold well, with sharp line widths, off-diagonal peaks for the top and bottom helices, and a characteristic peak at ∼10.6 ppm consistent with the formation of the GNRA tetraloop (Figure 7E). Both ^1^H–^13^C HSQC and ^1^H–^1^H D_2_O NOESY experiments were also completed on this construct (Figures 7E, F, S8, and Table S5), which revealed sharp, well resolved peaks indicating a stable single population. Taken together, the NMR data suggests that differences in neighboring base pairs of the 2 × 2 CC/CC internal loops give rise to very minor changes in structure. These minor changes in structure were not distinct enough to discern unique structural differences in 2D ^1^H–^13^C HSQC, ^1^H–^1^H H_2_O NOESY or ^1^H–^1^H D_2_O NOESY spectra (Figures 7, and S7 and S8).

**Figure 8.**
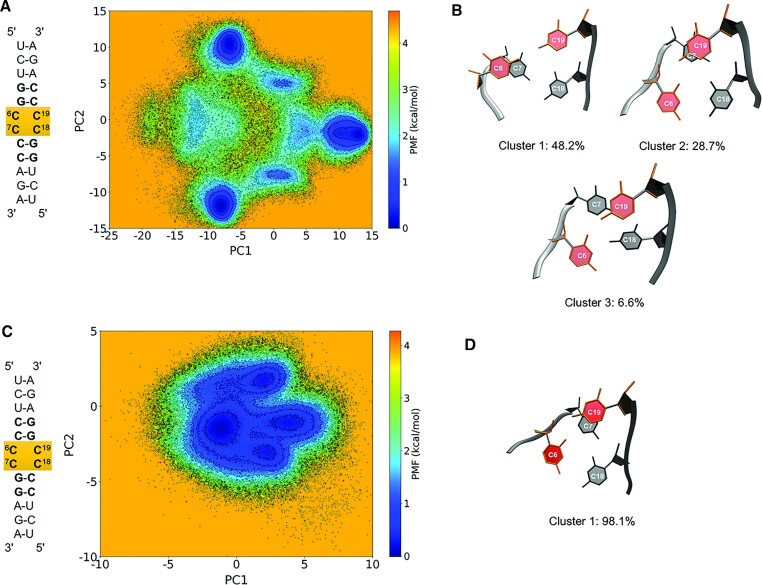
Properties of 2 × 2 CC/CC internal loops observed in r(G_2_C_4_)^exp^. Free energy landscapes of 2 × 2 CC/CC loops in G_2_C_4_ (**A**) and C_4_G_2_ (**C**) along the PC1 and PC2 extracted from 70 μs long MD simulations at 300K. While G_2_C_4_ displays three distinct minima (**B**), 2 × 2 CC/CC loop in C_4_G_2_ exclusively prefers one conformational state (**D**). Note that conformations in B and D displays structural similarities such as formation of single hydrogen bond states in C/C mismatches and stacking in helical axis.

To gain further insight into the conformations adopted by the 2 × 2 CC/CC internal loops formed by r(G_2_C_4_)^exp^, we performed regular MD simulations on model RNA constructs that were run for 70 μs each followed by cluster, PCA, and free energy landscape analyses (Figure 8). Potential of mean force (PMF) is a very useful tool in studying free energy changes as a function of reaction coordinates especially in biological systems (70). We predicted the PC1 and PC2 of the loop and the immediate neighboring base-pairs, yielding four base-pairs in total, and used them as reaction coordinates to generate the free energy landscapes for these two constructs (Figure 8). PMF analyses with respect to PC1 and PC2 showed that the dynamics experienced in GCCC is different from CCCG. While GCCC displayed multiple distinct minima, CCCG showed just one distinct minimum (Figure 8). We further investigated the MD trajectories using cluster analyses and observed that the MD trajectory of GCCC can be clustered into two major and one minor clusters (Figure 8B), where either one or both of the C/C mismatches were forming a single hydrogen bond in these clusters. In CCCG construct, trajectory was clustered into one main cluster, where hydrogen bonding pattern was alike to GCCC (Figure 8D). Similar hydrogen bonding patters has been observed in X-ray crystal structures (30).

It is noteworthy to highlight that MD calculations predict atomistic details of 2 × 2 CC/CC internal loops where cluster analyses can resolve even small details, which are not always possible in NMR experiments. Even though MD calculations predict GCCC to have multiple different conformational states compared to CCCG, these structures display similar properties such as the loop residues preferring *anti* orientations, stacking in helical axis, and forming at least a single hydrogen bond state. As a result, NMR spectroscopy might not be able to distinguish these individual states displaying relatively similar characteristics. Furthermore, it is also possible that the RNA force fields are still not be able to properly represent the 2 × 2 CC/CC loops, which can explain the differences observed between the NMR data and MD predictions.

## DISCUSSION

Structure-based drug design is based on knowledge of a binding pocket located within a biological macromolecule, where recognition of small molecule is dictated by the physical interactions such as hydrophobic, electrostatic, and hydrogen bonding interactions. Depending on the complexity and dynamics of the target, different experimental techniques such as X-ray crystallography, NMR spectroscopy, and cryogenic electron microscopy (cryo-EM) can be used to discover the atomistic details of a structure. For example, 1 × 1 G/G internal loop dynamics have been recently studied by NMR spectroscopy, where it was shown that the location of neighboring GC base pairs can greatly influence the rate of *syn* ↔ *anti* base flipping in 1 × 1 internal loops in the context of r(G_4_C_2_) repeats (28). These studies highlighted that stacking interactions of closing base pairs have a critical contribution to neighboring RNA loop dynamics (28). The work corroborates the sensitivity of r(G_4_C_2_)^exp^ to stacking contributions, showing that internal loop dynamics can be altered by the identity of neighboring nucleotides. Importantly, these structural rearrangements are important in target recognition as shown in binding mode of DNA mismatch repair enzyme MutS with 1 × 1 G/G mismatches (71) and in discovery of biomarkers and lead compounds targeting r(G_4_C_2_)^exp^ (72).

In many cases, it is challenging for experimental approaches to provide the in-depth details of small molecule-RNA interactions, which can be investigated with computational methods instead. This approach is especially useful when investigating highly dynamic RNA structures, which can prefer multiple different conformational states. Even though complexity of an RNA structure presents challenges, it also provides unique opportunities in drug design, where one can capitalize specific conformational states to enhance specificity and sensitivity. RNA motifs such as internal loops can transiently switch between different conformational states introducing localized dynamics within RNA as shown here in 2 × 2 GG/GG and 2 × 2 CC/CC internal loops. Small molecules, which can recognize specific transient states, can be utilized to exploit these conformational states of RNA, which can be used to inhibit RNA-associated disease mechanisms. This approach has the advantage to target a specific RNA molecule while avoiding other cellular RNAs, which is crucial in drug design as shown in HIV-1 stem–loop RNAs (73). Nonspecific binding, which is closely related to electrostatic (74,75) and stacking interactions (76,77), cause many side effects when tested clinically (78,79). Despite recent efforts on designing drug-like small molecules while overcoming non-specific binding to RNAs (21,80,81), selectivity is still a challenge. Design of small molecules, which can stabilize transient RNA conformations, can improve selectivity and provide a solution to nonspecific binding. Indeed, many regulatory RNAs can form low populated conformations, which display alternate base pairings especially in dynamic RNA loop motifs such as internal and bulge loops (7,82). Minor populations have been observed in riboswitches (8,12,83) and HIV-1 transactivation response element (TAR) (7) that has important implications in recognition, which provide selectivity upon ligand binding. In the case herein with 2 × 2 GG/GG internal loops, NMR studies and MD simulations delineated two main conformations that are dynamically interconverting. It is worth noting that the conformational slipping observed herein likely reflect both kinetic and thermodynamic contributions. Such contributions could be further explored by computational studies by completing either a large number of short simulations and an estimation of kinetics with a Markov state model or by using a bias potential facilitating rare events and then estimating the unbiased thermodynamics with reweighting estimators.

As described above, capitalization of these low abundant RNA conformations with small molecules could provide a novel approach in RNA therapeutics for currently incurable diseases (11,84–86). Thus, structural knowledge of such short-lived conformations is pivotal to utilize this new approach so that one can make use of transient RNA states in drug design. In this contribution, we combined advanced computational techniques with NMR spectroscopy to provide structural insights on highly dynamic 2 × 2 GG/GG and 2 × 2 CC/CC internal loop motifs observed in RNA G_4_C_2_ and G_2_C_4_ repeat expansions causing c9ALS/FTD. Results will pave the way for conformation specific small molecule design to modify RNA function in cell and to correct currently untreatable diseases.

## DATA AVAILABILITY

All data is contained within the manuscript and/or supplementary files.

## Supplementary Material

gkad403_Supplemental_Files
