# Supplementary material for: Conformational dynamics of RNA G4C2 and G2C4 repeat expansions causing ALS/FTD using NMR and molecular dynamics studies
Source: Nucleic Acids Res. 2023 May 22;51(11):5325–40. doi: 10.1093/nar/gkad403 (PMC10287959; doi:10.1093/nar/gkad403)
Supplement: gkad403_Supplemental_Files [file gkad403_supplemental_files.zip › Taghavi et al SI.pdf]

Supplemental Information for:

Conformational Dynamics of RNA G_4C_2 and G_2C_4 Repeat Expansions causing ALS/FTD using NMR and Molecular Dynamics Studies

Amirhossein Taghavi[1,†], Jared T. Baisen[1,†], Jessica L. Childs-Disney[1], Ilyas Yildirim[2,*], and Matthew D. Disney[1,*]

[1] Department of Chemistry, UF Scripps Biomedical Research, 130 Scripps Way, Jupiter, FL 33458 USA
[2] Department of Chemistry and Biochemistry, Florida Atlantic University, Jupiter, FL 33458 USA

[†] These authors contributed equally.
[*] Authors to whom correspondence is addressed; Email: iyildirim@fau.edu (I.Y.), disney@scripps.edu (M.D.D).

Table of Contents

**Movie S1.**	MD trajectories of r(G_4C_2)$_2$ predicted by four different RNA force fields to validate strand slippage.	**p. S3**
**Table S1.**	Sample input files used in minimization, equilibration, MD and T-REMD runs.	**p. S4**
**Table S2.**	1x1 G/G internal loops in literature.	**p. S5-7**
**Table S3.**	1H NMR chemical shifts of G_4C_2 RNA construct.	**p. S8**
**Table S4.**	1H NMR chemical shifts of C_4G_2 RNA construct.	**p. S9**
**Table S5.**	1H NMR chemical shifts of G_2C_4 RNA construct.	**p. S10**
**Script S1.**	In-house code to perform cluster analyses.	**p. S11-S15**
**Script S2.**	Convergence analyses performed on REMD trajectory.	**p. S16-17**
**Script S3.**	Principal Component and Kullback-Leibler divergence analysis.	**p. S18-19**
**Figure S1.**	Kullback−Leibler divergence (KLD) analyses.	**p. S20**
**Figure S2.**	Dilution and initial 1D NMR characterization of model G_4C_2 construct.	**p. S21**
**Figure S3.**	NMR characterization of non-exchangeable protons in G_4C_2.	**p. S22-23**
**Figure S4.**	NMR Characterization of exchangeable protons at lower temperature in G_4C_2.	**p. S24**
**Figure S5.**	NMR characterization of exchangeable protons in m^1G RNA constructs.	**p. S25-26**
**Figure S6.**	Influence of neighboring nucleotides on internal loop.	**p. S27**
**Figure S7.**	NMR characterization of non-exchangeable protons in C_4G_2.	**p. S28**
**Figure S8.**	NMR characterization of non-exchangeable protons in G_2C_4.	**p. S29**

Movie S1. MD trajectories of r(G_4C_2)$_2$ predicted by four different RNA force fields to validate strand slippage mechanism upon 2×2 GG/GG → 1×1 G/G transformations. The 2×2 GG/GG loop residues are highlighted in red/yellow colors to underline their behavior for the duration of the simulation. For detailed explanation of the observed transformations refer to the main text.

Table S1. Sample input files used in minimization, equilibration, MD and T-REMD runs.

```
Minimization of RNA with positional restraints – First Step
&cntrl
  imin=1, maxcyc=5000, ncyc=2500, ntb=1, cut= 10.0,
  ig=-1, ntr=1, ntp=0, ntf=1,
  restraintmask=":1-764 & !@H=", restraint_wt=10.0,
  ntxo=2, ioutfm=1,
/
```

```
Minimization of RNA without any restraints – Second Step
&cntrl
  imin=1, maxcyc=5000, ncyc=2500, ntb=1, cut= 10.0,
  ig=-1, ntr=0, ntp=0, ntf=1,
  ntxo=2, ioutfm=1,
/
```

```
NVT heating up the system from 0 to 50 with positional restraints
&cntrl
  imin=0, ntpr=1000, ntwr=5000, ntwx=1000, irest=0, ntx=1,
  ntb=1, cut=10.0, ntc=2, ntt=3, tempi=0.0, temp0=50.0,
  ntf=2,
  gamma_ln=1.0, nstlim=250000, dt=0.002, ntxo=2,
  ig=-1, ntr=1, restraint_wt=10.0, restraintmask=":1-764 & !@H=",
  ntxo=2, iwrap=1, ioutfm=1
/
```

```
NPT running with positional restraints
&cntrl
  imin=0, ntpr=5000, ntwr=5000, ntwx=5000, irest=1, ntx=5,
  ntb=2, cut=10.0, ntc=2, ntt=3, tempi=300.0, temp0=300.0,
  ntf=2, ntp=1, barostat=1, taup=2.0, pres0 = 1.0,
  gamma_ln=1.0, nstlim=1000000, dt=0.002, ntxo=2,
  ig=-1, ntr=1, restraint_wt=10.0, restraintmask=":1-764 & !@H=",
  ntxo=2, iwrap=1, ioutfm=1
/
```

```
T-REMD input file running at T = 364.5 K
&cntrl
  imin=0, ntpr=1000, ntwr=100000, ntwx=50000, irest=1, ntx=5,
  ntb=1, cut=8.0, ntc=2, ntt=3, temp0=364.5,
  ntf=2,
  gamma_ln=1.0, nstlim=5000, dt=0.002, ntxo=2,
  ig=-1, ntxo=2, iwrap=1, ioutfm=1,
  numexchg=15000,
/
```

Table S2. 1x1 G/G internal loops in literature. PDB entries displaying 1x1 G/G internal loops extracted from CoSSMos database show that possible conformations adopted by 1x1 G/G as 55% *anti-syn*, 16% *syn-anti*, and 26% *anti-anti*.

PDB ID	Nucleic Acid Type	Motif Sequence	Orientation<br>5′->3′<br>3′->5′
1F5G	RNA	CAGGC<br>GUGCG	*anti-syn*
1F5H	RNA	CAGGC<br>GUGCG	*syn-anti*
2L3C	HYDROLASE/RNA	UAGUA<br>AUGAU	*anti-syn*
2L3J	HYDROLASE/RNA	UAGUA<br>AUGAU	*anti-syn*
2NCQ	RNA	GCGGC<br>CGGCG	*syn-anti*
2NCR	RNA	GCGGC<br>CGGCG	*anti-anti*
3J80	RIBOSOME	CUGUG<br>GAGAU	*anti-syn*
3JAM	TRANSLATION	CUGUG<br>GAGAU	*anti-syn*
3JAP	TRANSLATION	CUGUG<br>GAGAU	*anti-syn*
3JAQ	TRANSLATION	CUGUG<br>GAGAU	*anti-syn*
3SJ2	RNA	CCGGC<br>GGGCG	*syn-anti*
3W3S	LIGASE/RNA	AGGUC<br>UCGAG	*syn-anti*
4D5L	RIBOSOME	ACGAA<br>UGGUU	*anti-anti*
4D61	RIBOSOME	ACGAA<br>UGGUU	*anti-anti*
4E5C	RNA	GCGGC<br>CGGCG	*anti-syn*
4KQ0	RNA BINDING PROTEIN/RNA	GCGGC<br>CGGCG	*anti-syn*
4KTG	RNA BINDING PROTEIN	CGGCG<br>GCGGC	*syn-anti*

Table S2. *Continued.*

4KTG	RNA BINDING PROTEIN	CGGCG<br>GCGGC	*anti-syn*
4KZX	RIBOSOME	ACGAA<br>UGGUU	*anti-anti*<br>*(G1377-G1344)*
4KZX	RIBOSOME	ACGAA<br>UGGUU	*anti-syn*<br>*(G153-G165)*
4R0D	RNA	GCGUA<br>UGGAU	*syn-anti*
4UER	TRANSLATION	ACGAA<br>UGGUU	*anti-anti*<br>*(G1324-G1291)*
4UER	TRANSLATION	ACGAA<br>UGGUU	*anti-syn*<br>*(G151-G163)*
4WCE	RIBOSOME	AGGGC<br>UCGCG	*anti-syn*
4WF9	RIBOSOME	AGGGC<br>UCGCG	*anti-syn*
4WFA	RIBOSOME	AGGGC<br>UCGCG	*anti-syn*
4WFB	RIBOSOME	AGGGC<br>UCGCG	*anti-syn*
5A2Q	RIBOSOME	ACGAA<br>UGGUU	*anti-syn*
5FLX	RIBOSOME	ACGAA<br>UGGUU	*anti-anti*
5HKV	RIBOSOME	AGGGC<br>UCGCG	*anti-syn*
5HL7	RIBOSOME	AGGGC<br>UCGCG	*anti-syn*
5JPQ	RIBOSOME	CUGUG<br>GAGAU	*anti-anti*
5K0Y	TRANSLATION	ACGAA<br>UGGUU	*anti-anti*
5LL6	RIBOSOME	CUGUG<br>GAGAU	*anti-syn*
5NRG	RIBOSOME	AGGGC<br>UCGCG	*anti-anti*
5OA3	TRANSLATION	ACGAA<br>UGGUU	*anti-syn*

Table S2. *Continued.*

5TZS	TRANSLATION	CUGUG<br>GAGAU	*anti-anti*
5UNE	RNA	AUGGU<br>UAGUA	*anti-anti*
5XXU	RIBOSOME	ACGAA<br>UGGUU	*anti-anti*
6CHR	RNA	GCGUA<br>UGGAU	*syn-anti*
6DDD	RIBOSOME	AGGGC<br>UCGCG	*anti-syn*
6DDG	RIBOSOME	AGGGC<br>UCGCG	*anti-syn*
6EML	RIBOSOME	ACGAA<br>UGGUU	*anti-anti*
6FAI	RIBOSOME	ACGAA<br>UGGUU	*syn-anti*
6G18	RIBOSOME	ACGAA<br>UGGUU	*anti-syn*
6G5H	RIBOSOME	ACGAA<br>UGGUU	*anti-syn*
6G5I	RIBOSOME	ACGAA<br>UGGUU	*anti-syn*
6HMA	RIBOSOME	AGGGC<br>UCGCG	*anti-syn*
6ME0	RNA	CAGGU<br>GUGCG	*anti-syn*
6P4G	RIBOSOME	ACGAA<br>UGGUU	*anti-syn*
6P4H	RIBOSOME	ACGAA<br>UGGUU	*anti-syn*
6PPK	RIBOSOME	AGGGC<br>UCGCG	*anti-anti*
6RA4	RNA BINDING PROTEIN	GUGAC<br>CAGUG	*syn-anti*
6RBD	RIBOSOME	CUGUG<br>GAGAU	*anti-syn*
6RBE	RIBOSOME	ACGAA<br>UGGUU	*anti-anti*

Table S3. ^{1}H NMR chemical shifts of the r(G_4C_2) RNA construct (Figure S2A). Non-exchangeable protons were assigned at 25˚C. Exchangeable protons were assigned at 10˚C.

Residue	H1 / H3	C6 / C8	H6/H8	C2	H5/H2	H1′	H2′	H3′
**C1**	-	140.56	8.105	**-**	5.97	5.493	4.34	4.45
**C2**	-	139.37	7.952	**-**	5.545	5.479	4.517	4.631
**A3**	-	136.66	7.971	149.8	6.924	5.815	4.451	4.114
**G4**	12.07	**B**	**B**	**-**	**-**	5.517	**B**	**B**
**G5**	**B**	**B**	**B**	**-**	-	**B**	**B**	**B**
**G6**	**B**	**B**	**B**	**-**	-	**B**	**B**	**B**
**C7**	-	**B**	**B**	-	-	5.42	**B**	**B**
**A8**	-	136.85	7.986	149.37	6.57	5.76	4.372	4.51
**A9**	-	136.02	7.704	150.53	7.251	5.785	4.24	4.576
**G10**	10.6	132.36	6.924	**-**	**-**	5.575	4.205	4.404
**G11**	13.03	133.43	7.07	**-**	**-**	5.583	4.256	4.507
**A12**	-	139.4	8.206	151.26	7.64	5.572	4.144	4.197
**A13**	-	137.25	7.829	151.51	7.537	5.34	4.274	4.527
**A14**	-	137.27	8.052	152.98	8.112	5.94	4.146	4.066
**C15**	-	139.21	7.815	**-**	5.956	3.451	4.175	3.907
**U16**	13.82	139.65	7.86	**-**	5.358	5.447	4.36	4.454
**U17**	13.31	138.94	7.945	**-**	5.542	5.489	4.518	4.073
**G18**	12.17	**B**	**B**	**-**	**-**	5.63	**B**	**B**
**G19**	**B**	**B**	**B**	**-**	-	**B**	**B**	**B**
**G20**	**B**	**B**	**B**	**-**	-	**B**	**B**	**B**
**C21**	-	**B**	**B**	**-**	5.08	5.46	4.236	4.43
**U22**	13.36	138.74	7.897	**-**	5.384	5.416	4.041	4.533
**G23**	13.3	133.13	7.538	**-**	-	5.695	4.388	4.483
**G24**	12.82	134.2	7.175	**-**	**-**	5.668	3.875	4.085

Table S4. ^{1}H NMR chemical shifts of the r(C_4G_2) RNA construct (Figure S6A). Non-exchangeable protons were assigned at 25˚C. Exchangeable protons were assigned at 5˚C.

Residue	H1 / H3	C6 / C8	H6/H8	C2	H5/H2	H1′	H2′	H3′
**C1**	-	140.74	8.101	**-**	5.991	**B**	4.45	4.356
**C2**	-	139.13	7.971	**-**	5.588	5.48	4.414	3.862
**A3**	-	136.88	8.094	151.11	7.418	5.894	3.996	4.346
**C4**	-	137.59	7.367	**-**	5.267	5.29	4.199	4.103
**C5**	-	139.51	7.795	**-**	5.666	5.429	3.934	4.372
**C6**	-	141.01	7.872	**-**	5.797	5.353	4.257	4.469
**G7**	11.99	134.25	7.671	**-**	-	5.7	4.591	4.393
**A8**	-	137.13	7.668	150.41	7.036	5.884	4.446	4.572
**A9**	-	136.2	7.643	150.41	7.356	5.872	4.44	4.53
**G10**	12.98	132.48	7.085	**-**	-	5.672	4.299	4.353
**G11**	10.62	133.2	7.127	**-**	-	5.635	4.279	4.526
**A12**	-	139.55	8.246	151.04	7.661	5.588	4.148	4.196
**A13**	-	137.39	7.875	151.47	7.573	5.351	4.293	4.535
**A14**	-	137.38	8.094	152.85	8.126	5.942	4.361	4.07
**C15**	-	139.22	7.801	**-**	5.963	3.453	4.163	3.921
**U16**	13.91	139.68	7.898	**-**	5.371	5.48	4.313	4.468
**U17**	13.09	140.14	7.986	**-**	5.492	5.559	4.385	4.472
**C18**	-	138.7	7.67	**-**	5.543	5.406	4.271	4.154
**C19**	-	139.26	7.821	**-**	5.666	5.292	3.91	4.389
**C20**	-	140.99	7.898	**-**	5.798	5.309	4.067	4.463
**G21**	12.19	134.5	7.717	**-**	-	5.623	4.387	4.499
**U22**	13.06	139.57	7.63	**-**	4.986	5.568	4.071	4.178
**G23**	12.93	133.41	7.641	**-**	**-**	5.761	4.467	4.43
**G24**	**B**	**B**	**B**	**-**	**-**	5.714	4.922	4.126

Table S5. ^{1}H NMR chemical shifts of r(G_2C_4) RNA construct (Figure S7A). Non-exchangeable protons were assigned at 25˚C. Exchangeable protons were assigned at 5˚C.

Residue	H1 / H3	C6 / C8	H6/H8	C2	H5/H2	H1′	H2′	H3′
**C1**	-	140.6	8.087	**-**	5.967	5.482	4.355	4.431
**C2**	-	138.9	7.92	**-**	5.555	5.475	4.431	4.622
**A3**	-	136.88	8.018	150.31	7.149	5.909	4.265	4.428
**G4**	12.13	132.57	6.894	**-**	**-**	5.31	3.952	4.468
**C5**	-	138.97	7.535	**-**	5.27	**B**	4.121	4.311
**C6**	-	140.89	7.941	**-**	5.773	**B**	3.837	4.474
**C7**	-	139.73	7.826	-	5.712	5.605	4.516	4.332
**A8**	-	137.39	7.999	149.61	6.649	5.822	4.547	4.366
**A9**	-	136.27	7.723	150.43	7.301	5.855	4.435	4.573
**G10**	12.97	132.45	7.078	**-**	**-**	5.646	4.275	4.348
**G11**	10.58	133.19	7.112	**-**	**-**	5.62	4.356	4.518
**A12**	-	139.59	8.248	150.93	7.665	5.59	4.146	4.193
**A13**	-	137.43	7.87	151.39	7.565	5.355	4.339	4.526
**A14**	-	137.38	8.064	152.74	8.124	5.947	4.057	4.152
**C15**	-	139.29	7.811	**-**	5.949	3.506	4.192	3.907
**U16**	13.91	139.58	7.848	**-**	5.355	5.478	4.333	4.463
**U17**	12.54	139.31	7.896	**-**	5.473	5.54	4.539	4.068
**G18**	12.33	133.58	7.611	**-**	**-**	5.631	4.5	4.327
**C19**	-	139.89	7.584	**-**	5.401	5.265	4.089	3.987
**C20**	-	141.19	7.892	**-**	5.859	**B**	**B**	**B**
**C21**	-	139.79	7.951	**-**	5.854	5.507	4.244	4.412
**U22**	12.71	139.34	7.686	**-**	5.377	5.478	4.081	4.43
**G23**	13.18	133.58	7.691	**-**	**-**	5.745	4.454	4.271
**G24**	12.13	134.26	7.273	**-**	**-**	5.71	3.927	4.126

Script S1. In-house code to perform cluster analyses, which incorporates symmetry of the system. See comment sections to get more information about the code.

```
#!/usr/bin/perl -w
#
# This uses linux commands to accelerate the process. Only 2 symmetric states are available. A
version of the trajectory where the atom locations are changed is created - again -
# to accelerate the calculations. Note also the cpptraj.MPI is faster when rmsd calculations are
performed.
#
# This script will uniquely cluster the conformations observed in MD trajectory. It uses other
perl script (create_symmetries.pl) to first create all the possible
# symmetric structures. These structures are then used to calculate the rmsd. The lowest rmsd
structures are then determined and clustered. Initially, the cutoff for
# rmsd is set to 1.0 Ang, which can be changed by user.
#
$mdcrd  = "./md.comb.nstep_10.nowat.traj";              # Trajectory file we are analyzing.
$mdcrds = "./md.comb.nstep_10.nowat.sym.traj";
$step  = 100;                           # This is the step we will use to extract rst data from
mdcrd, which will be used to calculate the rmsd values.
$cutoff = 1;                            # Cutoff for rmsd.
$refpdb = "reference.pdb";              # Reference .pdb file (which is used to create symmetric
states).
$prmtop = "strip.prmtop";               # Prmtop file
$pcheckf = "ptraj_check.in";            # Dummy ptraj check file.
 Note that for this script to work, the current directory has to have t
                                        # two files: 1) reference.pdb, and 2) symmetry.txt.
$parallel = "mpirun -np 64 cpptraj.MPI";
#################################################################### Check out the size of the
mdcrd file. Also, create the netcdf format. We will use this format because it is much faster than
trajectory format
#
$netcdf = "$mdcrd";
$netcdfs = "$mdcrds";
print "$size_mdcrd\n";
# Also, check out the number of atoms in the $refpdb is the same as the number of atoms defined in
the $prmtop file.
#
$system_size_from_ref = `cat $refpdb | grep ATOM | wc | awk '{print \$1}'`;
chomp($system_size_from_ref);
$system_size_from_prmtop = `grep -A 2 \"%FLAG POINTERS\" $prmtop | tail -1 | awk '{print \$1}'`;
chomp($system_size_from_prmtop);
if($system_size_from_ref != $system_size_from_prmtop){
  print "System sizes defined in $refpdb and $prmtop do not match. Exit ...\n";
  die;
}
#
###################################################################$RMScompound="";        # This
is the compound's atom list we will use in RMSd calculation.
open(F2O_compound, "RMScompounds.txt") || die "cannot open RMScompounds.txt: $!";
while(<F2O_compound>){
  chomp($_);
  $RMScompound=$_;
}
close(F2O_compound) || die "cannot close RMScompounds.txt: $!";
#
#################################################################### So, we know what the size of
the mdcrd is. Now, create a loop to extract the .rst files iteratively.
```

```
# Once an .rst is extracted from $mdcrd, the symmetric states will be first created. Later, we
will calculate the rmsd.
#
$count = -1;    # This variable is to count/create the number of cluster sets. Every cluster
related array will use this number. Note that array index starts from 0!
#
LABEL_STEP:
for($i=$step; $i <= $size_mdcrd; $i+=$step){
  #
  # Check if this particular snapshot/step is already clustered.
  #
  if($count != -1){
    $val = `echo $i > structure_to_check.txt ; diff old_structures.txt structure_to_check.txt |
grep "^>" | awk 'BEGIN{s=0}{s++}END{print s}'`;
    chomp($val);
    if($val != 1){
      system("rm -f structure_to_check.txt");
      next LABEL_STEP;
    }
  }
  $count++;
  #
  # We will do two calculations; first we will calculate the average structure of the initial
cluster, and second we will finalize the clustering process.
  # First phase of clustering: Reference structure is the snapshot taken from the trajectory file,
and an initial clustering will be done. This will yield
  # an average structure, which we will use in the second phase of clustering process.
  #
  open(F2W, ">$pcheckf") || die "cannot open $pcheckf: $!";
  print F2W "reference $netcdf $i $i\n";
  print F2W "trajin $netcdf\n";
  print F2W "rms reference out rms \"$RMScompound\" \n";
  close(F2W) || die "cannot close $pcheckf: $!";
  #
  system("$parallel $prmtop <$pcheckf> out_rms");
  #
  open(F2W, ">$pcheckf") || die "cannot open $pcheckf: $!";
  print F2W "reference $netcdf $i $i\n";
  print F2W "trajin $netcdfs\n";
  print F2W "rms reference out rms.s \"$RMScompound\" \n";
  close(F2W) || die "cannot close $pcheckf: $!";
  #
  system("$parallel $prmtop <$pcheckf> out_rms_s");
  #
  system("rm $pcheckf out_rms out_rms_s");
  #
  `paste rms rms.s | grep -v "#" | awk -v cutoff="$cutoff"
'{if((\$2<=cutoff)||(\$4<=cutoff)){if(\$2<\$4){print \$1"\t"\$2"\t1"}else{print
\$1"\t"\$4"\t2"}}}' > tmp.rms_all`;
  #
  # We have calculated the rmsd for each symmetric states. Now combine them. Note that we will
also store the symmetry state in an array (for future use).
  #
  `cat tmp.rms_all | awk '{print \$1}' > new_structures.txt`;
  #
  if($count == 0){      # First time we are creating the clusters..
    `cp new_structures.txt new_structures_to_add.txt`;
    `cat new_structures_to_add.txt | awk '{l=l \$1" "}END{gsub(/ \$/,"",l); print l}' >
tmp.oneliner`;
```

```
    `cat tmp.rms_all | awk '{s++; if(s==1){k="cat tmp.oneliner"; l=""; k|getline l ; close(k);
c=split(l,a," ")}; b[\$1]=\$0}END{for(i=1; i<=c; i++){print b[a[i]]}}' > cluster_$count\.txt`;
  } else {
    # We now have some clusters which were created before. We need to check out the structures in
those clusters to make sure that we are not double counting the same structures.
    `diff old_structures.txt new_structures.txt | grep "^>" | awk '{print \$NF}' | sort -k1,1n  >
new_structures_to_add.txt`;
    `cat new_structures_to_add.txt | awk '{l=l \$1" "}END{gsub(/ \$/,"",l); print l}' >
tmp.oneliner`;
    `cat tmp.rms_all | awk '{s++; if(s==1){k="cat tmp.oneliner"; l=""; k|getline l ; close(k);
c=split(l,a," ")}; b[\$1]=\$0}END{for(i=1; i<=c; i++){print b[a[i]]}}' > cluster_$count\.txt`;
  }
  $cluster_size = `cat tmp.oneliner | awk '{print NF}'`;
  chomp($cluster_size);
  #
  print "Step = $i\tCluster $count size = $cluster_size - Before convergence is started\n";
  #
  # This is a new section for the code. Previously, the structures in the trajectory are used in
clustering process. It is possible that sometimes that structure
  # cannot be a good comparison. Thus, we will first find the average structure of this new
cluster, and then re-do the cluster analyses. This way, structures missed
  # in the first clustering process will be included.
  #
  # First, combine the cluster, and find the average structure. The data will be located in the
cluster_$count directory.
  #
  system("./cluster_mdcrd_extract.sh cluster_$count\.txt");     # Note that the cluster_$count
directory created during this process is not the final version!
  system("cp cluster_$count/avg.rst ./; rm -dfr cluster_$count");
  #
  # We are in the second phase of the clustering where we will use the average structure as the
reference conformation during the clustering process.
  $flag_convergence = 0;
  $convergence_step = 1;
   while($flag_convergence == 0){
    #
    # We are in the second phase of the clustering where we will use the average structure as the
reference conformation during the clustering process.
    # We are going to compare it to new_structures_to_add.txt file
    #
    open(F2W, ">$pcheckf") || die "cannot open $pcheckf: $!";
    print F2W "reference avg.rst\n";
    print F2W "trajin $netcdf\n";
    print F2W "rms reference out rms \"$RMScompound\" \n";
    close(F2W) || die "cannot close $pcheckf: $!";
    system("$parallel $prmtop <$pcheckf> out_rms");
    #
    open(F2W, ">$pcheckf") || die "cannot open $pcheckf: $!";
    print F2W "reference avg.rst\n";
    print F2W "trajin $netcdfs\n";
    print F2W "rms reference out rms.s \"$RMScompound\" \n";
    close(F2W) || die "cannot close $pcheckf: $!";
    #
    system("$parallel $prmtop <$pcheckf> out_rms_s");
    system("rm $pcheckf out_rms out_rms_s");
    #
    `paste rms rms.s | grep -v "#" | awk -v cutoff="$cutoff"
'{if((\$2<=cutoff)||(\$4<=cutoff)){if(\$2<\$4){print \$1"\t"\$2"\t1"}else{print
\$1"\t"\$4"\t2"}}}' > tmp.rms_all`;
    #
```

```
    # We have calculated the rmsd for each symmetric states. Now combine them. Note that we will
also store the symmetry state in an array (for future use).
    #
    `cat tmp.rms_all | awk '{print \$1}' > new_structures.txt`;
    if($count == 0){    # First time we are creating the clusters..
      `cp new_structures.txt new_structures_to_add_conv-check.txt`;
      `cat new_structures_to_add_conv-check.txt | awk '{l=l \$1" "}END{gsub(/ \$/,"",l); print l}'
> tmp.oneliner`;
      `cat tmp.rms_all | awk '{s++; if(s==1){k="cat tmp.oneliner"; l=""; k|getline l ; close(k);
c=split(l,a," ")}; b[\$1]=\$0}END{for(i=1; i<=c; i++){print b[a[i]]}}' > cluster_$count\.txt`;
    } else {
      # We now have some clusters which were created before. We need to check out the structures
in those clusters to make sure that we are not double counting the same structures.
      `diff old_structures.txt new_structures.txt | grep "^>" | awk '{print \$NF}' | sort -k1,1n
> new_structures_to_add_conv-check.txt`;
      `cat new_structures_to_add_conv-check.txt | awk '{l=l \$1" "}END{gsub(/ \$/,"",l); print l}'
> tmp.oneliner`;
      `cat tmp.rms_all | awk '{s++; if(s==1){k="cat tmp.oneliner"; l=""; k|getline l ; close(k);
c=split(l,a," ")}; b[\$1]=\$0}END{for(i=1; i<=c; i++){print b[a[i]]}}' > cluster_$count\.txt`;
    }
    $diffset=`diff new_structures_to_add.txt new_structures_to_add_conv-check.txt | awk
'BEGIN{s=0}{s++}END{print s}'`;
    #
    if($diffset == 0){  # We are converged
      if($count == 0){
        `cp new_structures_to_add_conv-check.txt old_structures.txt; rm -f
new_structures_to_add_conv-check.txt`;
      } else {
        `cat old_structures.txt new_structures_to_add_conv-check.txt | sort -k1,1n > tmp.newset`;
        `cp tmp.newset old_structures.txt; rm -f tmp.newset new_structures_to_add_conv-check.txt`;
      }
      $cluster_size = `cat tmp.oneliner | awk '{print NF}'`;
      chomp($cluster_size);
      system("./cluster_mdcrd_extract.sh cluster_$count\.txt"); # Note that the cluster_$count
directory created during this process is not the final version!
      #
      print "Step = $i\tCluster $count size = $cluster_size - Final Version\n";
      system("rm -f tmp.oneliner new_structures_to_add.txt tmp.newset new_structures.txt
avg.rst");
      $flag_convergence = 1;    # Exit from the while loop
    } else {    # Not converged, do the same steps again using the new cluster_$count.txt set.
      $cluster_size = `cat tmp.oneliner | awk '{print NF}'`;
      chomp($cluster_size);
      print "Step = $i\tCluster $count size = $cluster_size - Convergence step
$convergence_step\n";
      system("./cluster_mdcrd_extract.sh cluster_$count\.txt"); # Redo the avg.pdb calculation
      system("cp cluster_$count/avg.rst ./; rm -dfr cluster_$count");
      system("cp new_structures_to_add_conv-check.txt new_structures_to_add.txt; rm
new_structures_to_add_conv-check.txt");
      $convergence_step++;
    }
  }
}
#
################################ Subroutines ################################
sub check_size {
  my($smdcrd) = (@_);
  open(SF20, ">$pcheckf") || die "cannot open $pcheckf: $!";
  print SF20 "trajin $smdcrd 1 1\n";    # Because this is for checking purposes, do not worry
about 1 1. Ptraj will print the size of the trajectory anyways...
```

```
  print SF2O "rms first :1\n";          # For sure there is at least one residue in the system, so
the script will work.
  close(SF2O) || die "cannot close $pcheckf: $!";
  system("cpptraj $prmtop <$pcheckf> out_check");
  my($s_size_mdcrd) = `cat out_check | grep \"reading 1\" | awk '{gsub(/\\)/,""); print \$NF}'`;
  chomp($s_size_mdcrd);
  return $s_size_mdcrd;
}
```

Script S2. Convergence analyses performed on REMD trajectory using clusters observed over 1%.

```
#!/bin/sh
#
# Will analyze the trajectory to determine the convergence. The trajectory is coming from REMD.
#
########################################################################
time=5000
limit=100000
#
# First, create a temp file to store the cluster_[0-9]*.txt file names
#
cat ../clusters_observerved_over_1_percentage.txt  | \
awk '{ \
  split($1,a,"_"); \
  l=l a[2] " "; \
} END { \
  gsub(/ $/,"",l); \
  print l \
}' > tmp.clusters.all
#
cat ../clusters_observerved_over_1_percentage.txt | \
awk '{ \
  print "# "$1; \
  system("cat ../"$1".txt") \
}' | \
awk '{ \
  if(/^#/){ \
    split($2,a,"_"); \
    cluster=a[2] \
  } else { \
    print $1"\t"$2"\t"cluster \
  } \
}' | sort -k1,1n | \
awk 'BEGIN{lb=0}{ \
  ub=$1; \
  diff=ub-lb; \
  if(diff != 1){ \
    for(i=lb+1; i<=ub-1; i++){ \
      print i \
    } \
  }; \
  print $0; \
  lb=ub \
}' | awk -v limit=$limit -v time=$time '{ \
  if($1 < limit){ \
    next; \
  } \
  c++; \
  if(NF == 1){ \
    d[c]="X"; \
  } else {
    d[c]=$NF; \
  }; \
} END { \
  k="cat tmp.clusters.all"; \
  l=""; \
  k | getline l; \
```

```
  close(k); \
  size=split(l,cluster," "); \
  for(i=1; i<=size; i++){ \
    t[cluster[i]]=0; \
  }; \
  unclustered=0; \
  for(i=1; i <= c-time; i++){ \
    for(j=i; j <= i + time; j++){ \
      if(d[j] == "X"){ \
        unclustered++; \
      } else { \
        t[d[j]]++; \
      } \
    } \
    l=i+limit-1"\t"; \
    for(j=1; j<=size; j++){ \
      l=l sprintf("%.2f ", t[cluster[j]]/time);
    } \
    print l; \
    for(j=1; j<=size; j++){ \
      t[cluster[j]]=0; \
    }; \
    unclustered=0; \
    l=""; \
  } \
}'
```

Script S3. Principal Component and Kullback-Leibler divergence analysis.

```
###########################################################
Principal Component/Kullback-Leibler Analysis
##########################################################
opt_2="6-7,18-19"
frame_1=$start1
frame_2=$end1
frame_3=$start2
frame_4=$end2
bin=300
cat >input<<EOF
parm ./strip.prmtop
trajin ./temp.302.comb.nc
# Average the trajectory
rms first :$opt_2&!@H=
average ggAvg.rst restart
# Save coordinates
run
# Read in average structure
reference ggAvg.rst [avg]
# RMS-fit coordinates in crd1 to average
crdaction crd1 rms ref [avg] :$opt_2&!@H=
# Calculate coordinate covariance matrix
crdaction crd1 matrix covar :$opt_2&!@H= name ggCovar
# Diagonalize coordinate covariance matrix for eigenmodes
runanalysis diagmatrix ggCovar out evecs.dat vecs 20
#create separate PC projections for each trajectory
crdaction crd1 projection T1 modes evecs.dat beg 1 end 20 :$opt_2&!@H= \
crdframes $frame_1,$frame_2 out T1.dat
crdaction crd1 projection T2 modes evecs.dat beg 1 end 20 :$opt_2&!@H= \
crdframes $frame_3,$frame_4 out T2.dat
# Calculate Kullback-Leibler Divergence vs time for PC histograms
# Trajectories 1 and 2, modes 1-5
kde T1:1 kldiv  T2:1 klout  KL-PC.${opt}.agr bins $bin name TREMD-1,PC1
kde T1:2 kldiv  T2:2 klout  KL-PC.${opt}.agr bins $bin name TREMD-2,PC2
kde T1:3 kldiv  T2:3 klout  KL-PC.${opt}.agr bins $bin name TREMD-3,PC3
kde T1:4 kldiv  T2:4 klout  KL-PC.${opt}.agr bins $bin name TREMD-4,PC4
kde T1:5 kldiv  T2:5 klout  KL-PC.${opt}.agr bins $bin name TREMD-5,PC5
kde T1:6 kldiv  T2:6 klout  KL-PC.${opt}.agr bins $bin name TREMD-6,PC6
kde T1:7 kldiv  T2:7 klout  KL-PC.${opt}.agr bins $bin name TREMD-7,PC7
kde T1:8 kldiv  T2:8 klout  KL-PC.${opt}.agr bins $bin name TREMD-8,PC8
kde T1:9 kldiv  T2:9 klout  KL-PC.${opt}.agr bins $bin name TREMD-9,PC9
kde T1:10 kldiv T2:10 klout KL-PC.${opt}.agr bins $bin name TREMD-10,PC10
# Calculate PC histogram with KDE, trajectory 1, mode 1
kde T1:1 out kde-PC.1.${opt}.agr bins $bin name KDE1-1
kde T1:2 out kde-PC.1.${opt}.agr bins $bin name KDE1-2
kde T1:3 out kde-PC.1.${opt}.agr bins $bin name KDE1-3
kde T1:4 out kde-PC.1.${opt}.agr bins $bin name KDE1-4
kde T1:5 out kde-PC.1.${opt}.agr bins $bin name KDE1-5
# Calculate PC histogram with KDE, trajectory 2, mode 1
kde T2:1 out kde-PC.2.${opt}.agr bins $bin name KDE2-1
kde T2:2 out kde-PC.2.${opt}.agr bins $bin name KDE2-2
kde T2:3 out kde-PC.2.${opt}.agr bins $bin name KDE2-3
kde T2:4 out kde-PC.2.${opt}.agr bins $bin name KDE2-4
kde T2:5 out kde-PC.2.${opt}.agr bins $bin name KDE2-5
# Calculate PC histogram, trajectory 1, mode 1
hist T1:1,*,*,*,$bin out pca.hist.1.${opt}.agr normint name HIST1-1
hist T1:2,*,*,*,$bin out pca.hist.1.${opt}.agr normint name HIST1-2
```

```
hist T1:3,*,*,*,$bin out pca.hist.1.${opt}.agr normint name HIST1-3
hist T1:4,*,*,*,$bin out pca.hist.1.${opt}.agr normint name HIST1-4
hist T1:5,*,*,*,$bin out pca.hist.1.${opt}.agr normint name HIST1-5
# Calculate PC histogram, trajectory 2, mode 1
hist T2:1,*,*,*,$bin out pca.hist.2.${opt}.agr normint name HIST2-1
hist T2:2,*,*,*,$bin out pca.hist.2.${opt}.agr normint name HIST2-2
hist T2:3,*,*,*,$bin out pca.hist.2.${opt}.agr normint name HIST2-3
hist T2:4,*,*,*,$bin out pca.hist.2.${opt}.agr normint name HIST2-4
hist T2:5,*,*,*,$bin out pca.hist.2.${opt}.agr normint name HIST2-5
EOF
cpptraj -i input
echo "cpptraj done!"
```

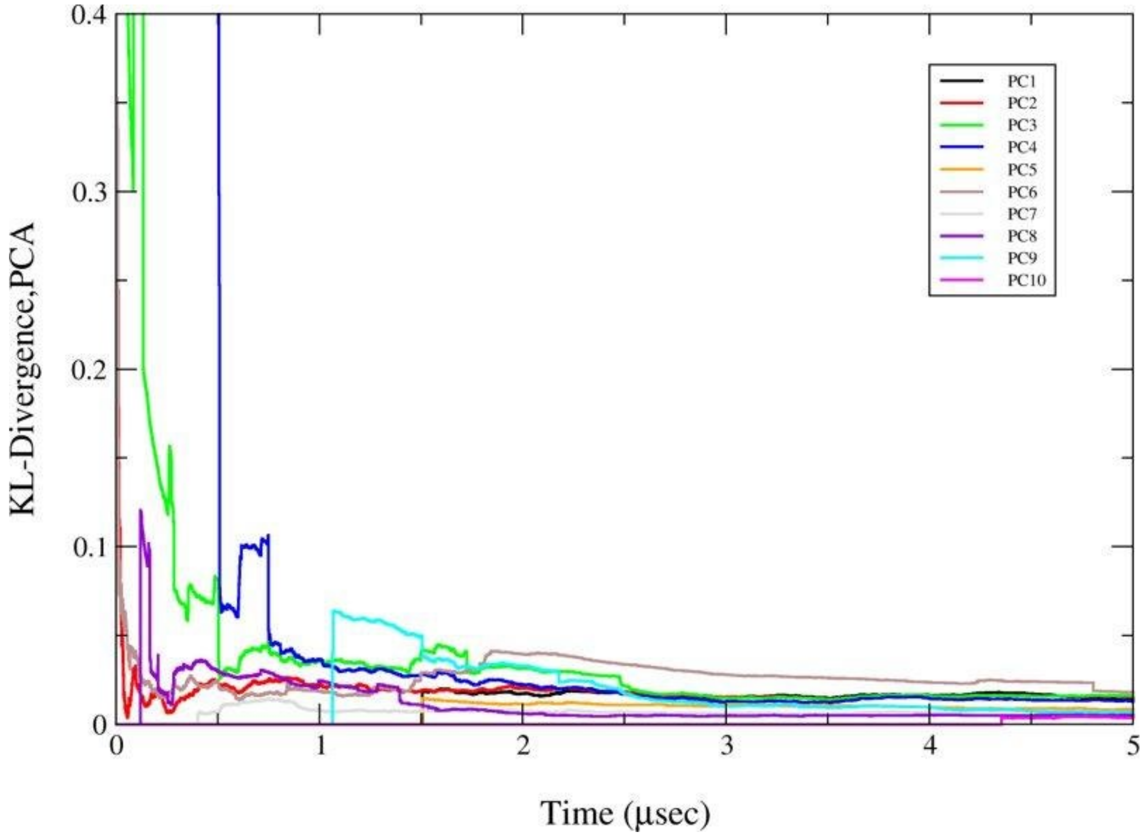

Figure S1. Kullback−Leibler divergence (KLD) of the histograms of the principal component projections for the first 10 principal components. Divergent analysis of the simulation time in the interval of 20-25 and 25-30 µsec of the first ten PCs show changes approaching to zero, but some still deviate.

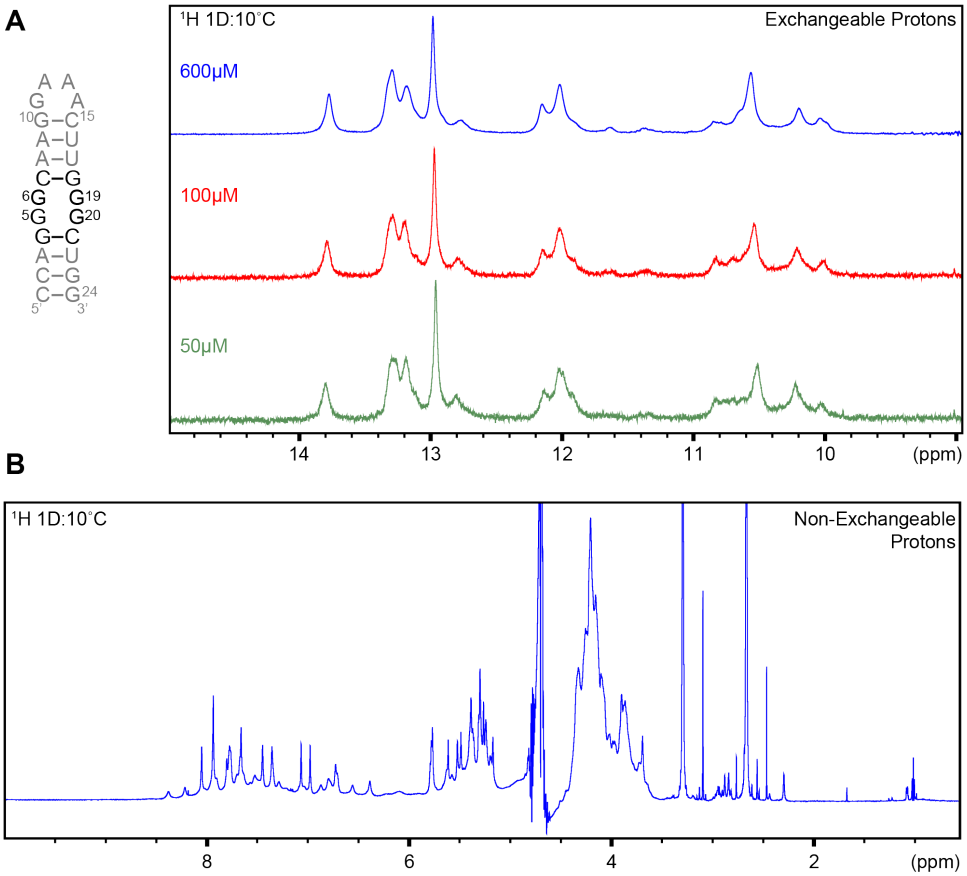

Figure S2. Initial 1D NMR spectroscopic characterization of a model r(G_4C_2) RNA construct. (A) Secondary structure of the model r(G_4C_2) construct along and ^{1}H 1D NMR spectrum of imino protons (H1/H3) with decreasing concentrations of the RNA. **(B)** Non-exchangeable ^{1}H 1D NMR proton spectrum for highest concentration sample (600 µM).

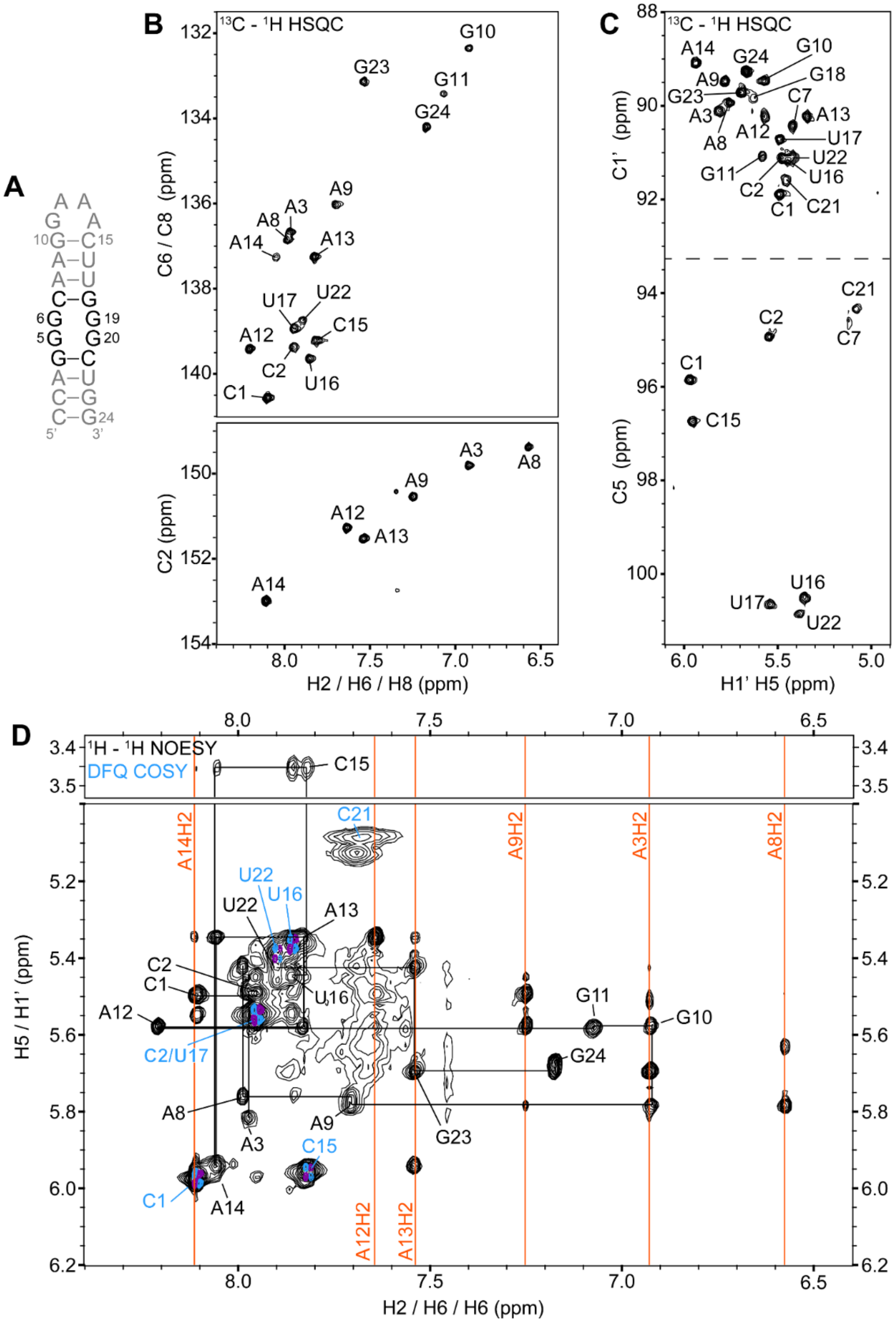

Figure S3. NMR Characterization of non-exchangeable protons in G_4C_2. (A) Secondary structure of the r(G_4C_2) construct (G<u>GG</u>C). **(B)** NMR ^{13}C-^{1}H HSQC spectra of aromatic (C2, C6, and C8) region, along with accompanying assignments. **(C)** NMR ^{13}C-^{1}H HSQC spectra of ribose (C1′) region, with cytosine and uridine aromatic (C5) region shown below. **(D)** ^{1}H–^{1}H 2D NOESY NMR spectra of "NOESY walk" (aromatic – ribose) region collected at 300ms mixing time, with DFQ COSY NMR spectra overlaid in cyan and purple, both collected at 25˚C.

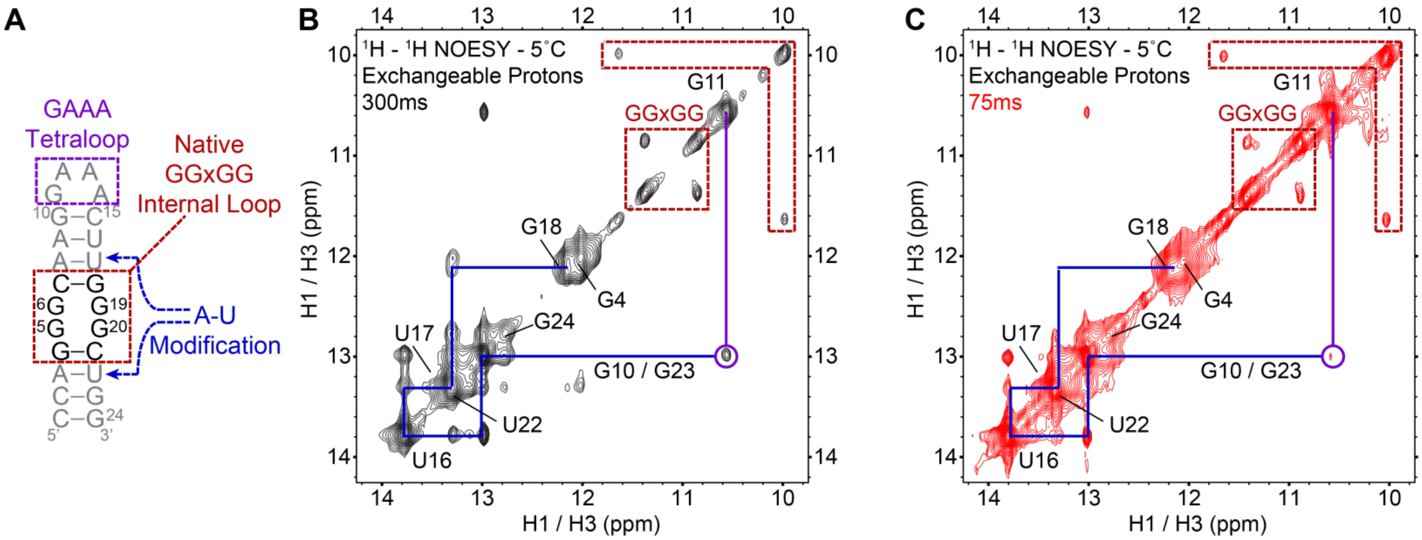

Figure S4. NMR spectroscopic characterization of exchangeable protons in G_4C_2 at 5°C. (A) Secondary structure of the model G_4C_2 construct. **(B, C)** ^{1}H-^{1}H Imino NOESY experiments for two mixing times at 5˚C. The GNRA tetraloop peak is highlighted by a purple line and circle. Additional off-diagonal peaks for the lower and upper stem are shown as blue lines. Non-canonical peaks associated with the 2re ×2 GG/GG shown as red dashed lines and lettering, while assignments for all other resonances are shown in grey.

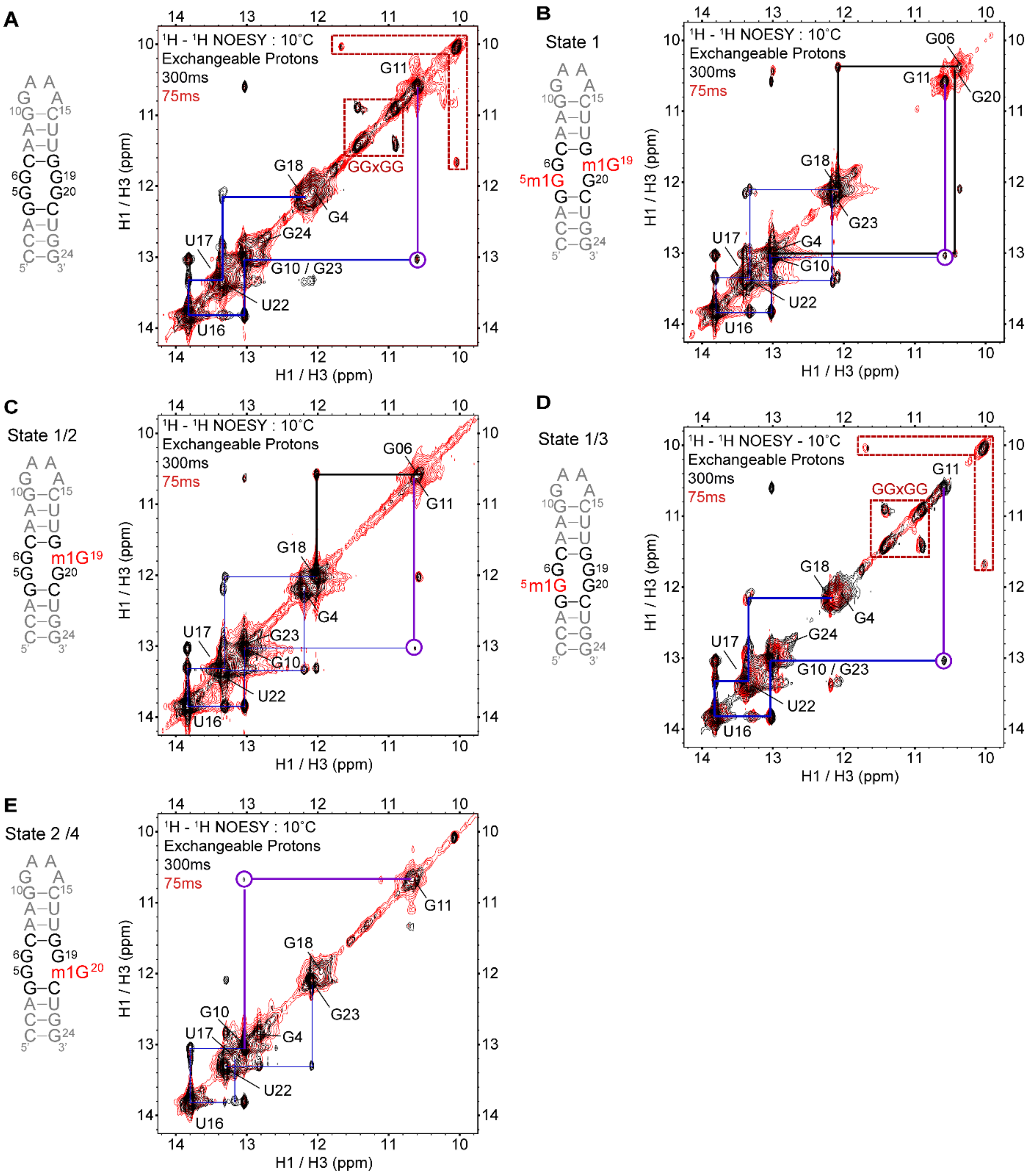

Figure S5. NMR characterization of exchangeable protons in m^{1}G RNA constructs. (A) Secondary structure of the model G4C2 construct along with ^{1}H-^{1}H Imino NOESY experiments for two mixing times at 10˚C. The GNRA tetraloop peak highlighted by a purple line and circle, additional off-diagonal peaks for the lower and upper stem shown as blue lines, non-canonical peaks associated with the 2×2 GG/GG shown as red dashed lines and lettering, and assignments for all other resonances shown in grey. (**B**) Secondary structure and accompanying ^{1}H-^{1}H Imino NOESY for G5/G19 doubly-modified NMR construct, G19 M1G construct (**C**), G5 M1G construct (**D**), and G20 M1G construct **(E)**, all shown with two mixing times, assignments, and non-canonical 2×2 GG/GG peaks highlighted with red dashed-lines and lettering.

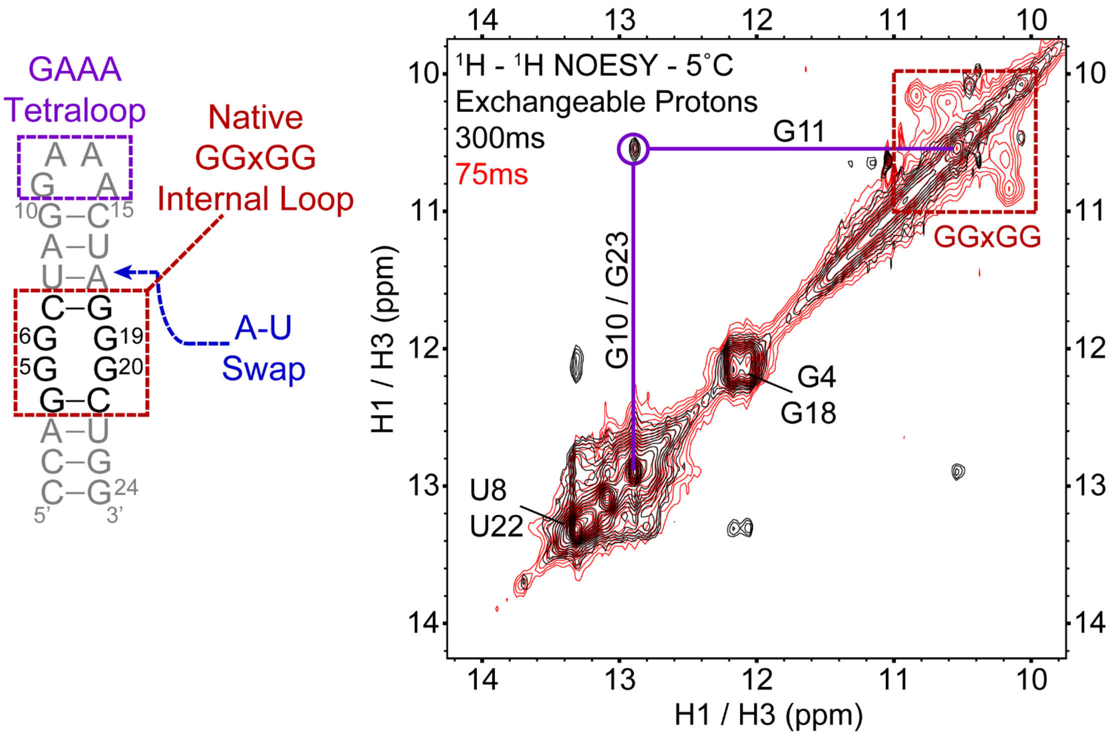

Figure S6. Neighboring nucleotides influence internal loop as characterized by NMR exchangeable protons. Secondary structure of an "A-U swap" modified model G_4C_2 RNA construct, with sequence modification to increase symmetry highlighted with a blue arrow (A8/U17 to U8/A17). Corresponding ^{1}H-^{1}H Imino NOESY spectra at two mixing times, collected at 10˚C. GNRA tetraloop peal highlighted by a line and circle, additional off-diagonal peaks for the lower and upper stem shown as grey lines, non-canonical peaks associated with the 2×2 GG/GG shown red dashed lines and lettering, and assignments for all other resonances shown in grey.

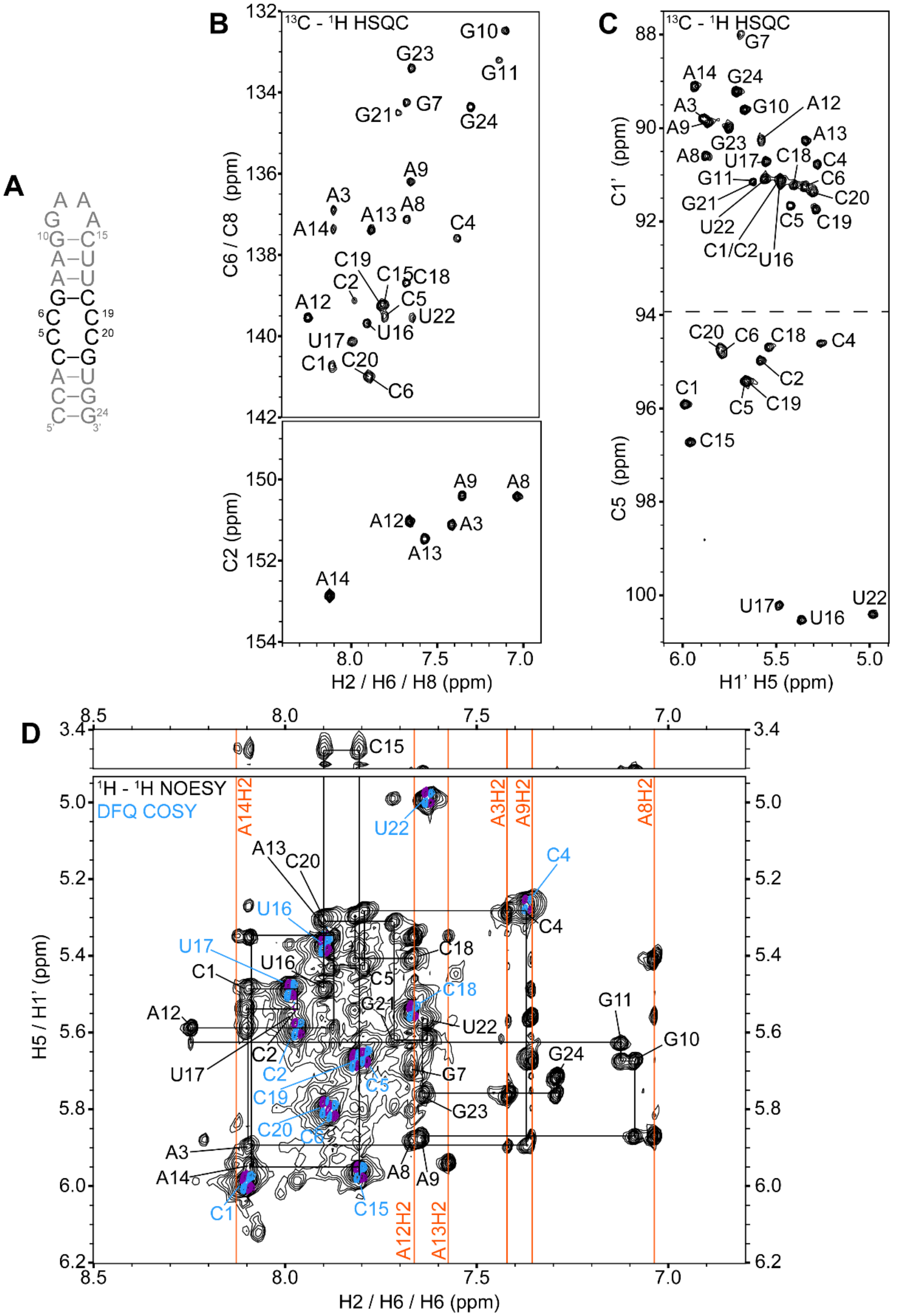

Figure S7. NMR Characterization of non-exchangeable protons in C$_4$G$_2$. (A) Secondary structure of the C$_4$G$_2$ construct (CCCG). **(B)** NMR ^{13}C - ^{1}H HSQC spectra of aromatic (C2, C6, and C8) region, along with accompanying assignments. **(C)** NMR ^{13}C-^{1}H HSQC spectra of ribose (C1′) region, with cytosine and uridine aromatic (C5) region shown below. **(D)** ^{1}H–^{1}H 2D NOESY NMR spectra of “NOESY walk” (aromatic – ribose) region collected at 300ms mixing time, with DFQ COSY NMR spectra overlaid in cyan and purple, both collected at 25˚C.

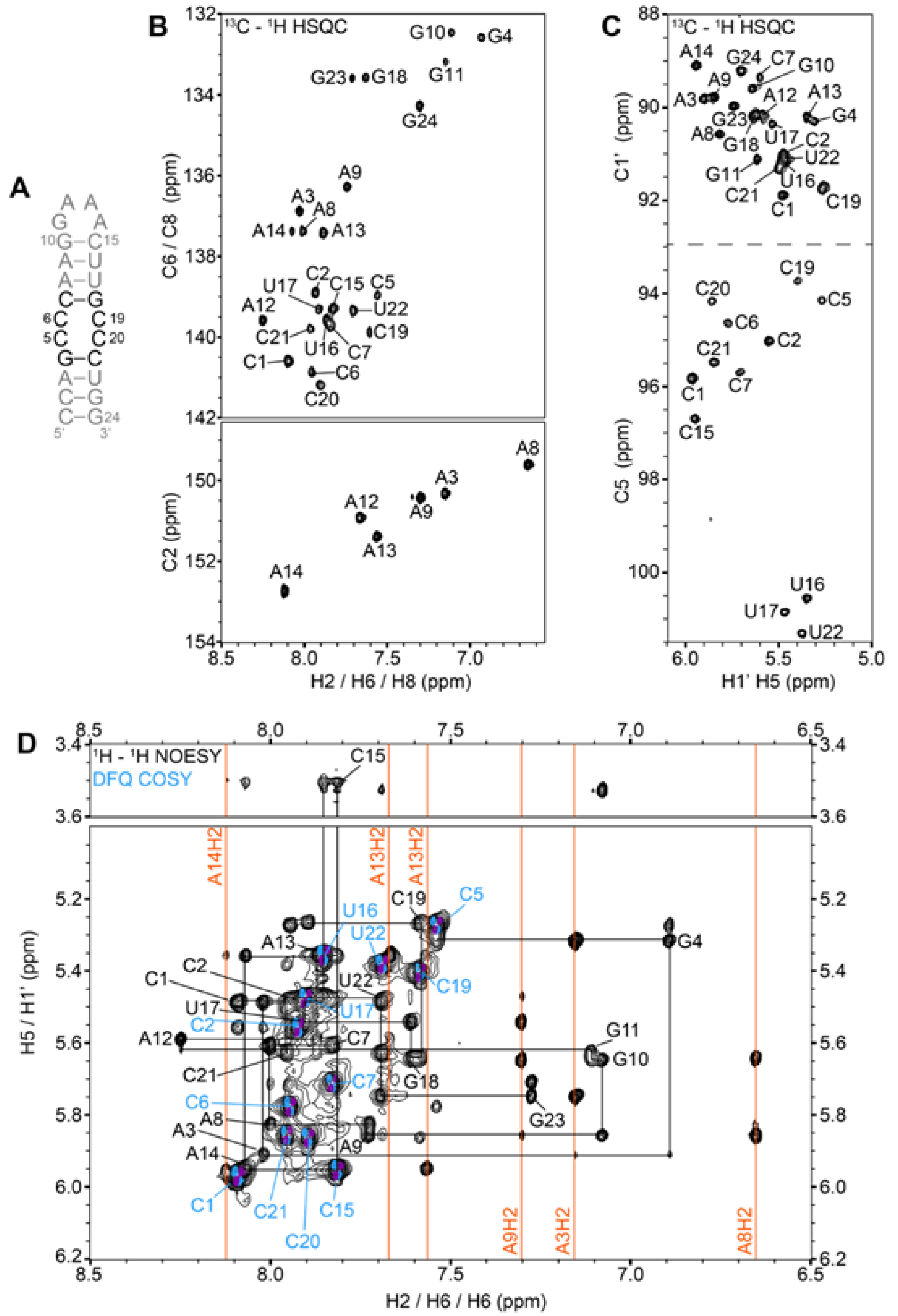

Figure S8. NMR Characterization of non-exchangeable protons in r(G_2C_4). (A) Secondary structure of the G_2C_4 construct (GCCC). **(B)** NMR ^{13}C-^{1}H HSQC spectra of aromatic (C2, C6, and C8) region, along with accompanying assignments. **(C)** NMR ^{13}C-^{1}H HSQC spectra of ribose (C1′) region, with cytosine and uridine aromatic (C5) region shown below. **(D)** ^{1}H–^{1}H 2D NOESY NMR spectra of "NOESY walk" (aromatic – ribose) region collected at 300ms mixing time, with DFQ COSY NMR spectra overlaid in cyan and purple, both collected at 25˚C.
